# Endophytic Fungus *Phomopsis liquidambaris* Enhances Fe Absorption in Peanuts by Reducing Hydrogen Peroxide

**DOI:** 10.3389/fpls.2022.872242

**Published:** 2022-04-29

**Authors:** Ying-Chun Du, Ling-Jie Kong, Ling-Sen Cao, Wei Zhang, Qiang Zhu, Chen-Yu Ma, Kai Sun, Chuan-Chao Dai

**Affiliations:** Jiangsu Key Laboratory for Microbes and Functional Genomics, Jiangsu Engineering and Technology and Research Center for Industrialization of Microbial Resources, College of Life Sciences, Nanjing Normal University, Nanjing, China

**Keywords:** endophytic fungus, iron deficiency, hydrogen peroxide, peanut, stress mitigation, transcriptome

## Abstract

Iron (Fe) deficiency in alkaline calcium soil is a problem that needs to be solved urgently as Fe is an essential and commonly limiting nutrient for plants. Endophytic fungus, *Phomopsis liquidambaris* (*P. liquidambaris*), has been reported to promote Fe absorption in peanuts (*Arachis hypogaea* L.), however, the mechanisms remain unclear. Under prolonged Fe deficiency, an increase in hydrogen peroxide (H_2_O_2_) often triggers a series of signaling events and leads to the inhibition of Fe acquisition. The main purpose of this study was to explore whether and how the endophytic fungus *P. liquidambaris* promote Fe absorption in peanut through regulating H_2_O_2_ and assisting in resisting oxidative stress. In this study, we detected the Fe deficiency-induced transcription factor (*FIT*), Fe^2+^ transporter (*IRT*1), and ferric reduction oxidase 2 (*FRO2*) of peanuts, and confirmed that they were negatively related to Fe concentration. Similarly, *FIT, IRT*1, and *FRO2* were also inhibited by H_2_O_2_. The addition of *P. liquidambaris* reduces H_2_O_2_ under Fe-deficiency with an increase in Fe content, while the exogenous addition of H_2_O_2_ further decreases it, and the addition of catalase (CAT) under Fe-deficiency reverses this phenomenon. Through transcriptome analysis, we proved that the expression of *FIT, IRT*1, *FRO2* and CAT are consistent with our hypothesis, and *P. liquidambaris* has a stress-mitigating effect on peanuts mainly via CAT, glutathione peroxidase, and malondialdehyde. Our study proved the Fe-absorption promoting effect and stress mitigation effect of *P. liquidambaris* under Fe-deficiency in peanuts, and their combined usage may help peanuts grow better

## Introduction

Iron (Fe) has redox properties, it is involved in key processes such as photosynthesis, chlorophyll biosynthesis, and electron transport, and is an essential nutrient for plants (Balk and Schaedler, 2014). Although Fe is the fourth most abundant element in the Earth's crust, it is not readily available to plants as it usually existed in the form of scarcely soluble Fe^3+^ oxyhydroxides, especially in alkaline calcareous soils (Abadia et al., 2011; Arikan et al., 2018). Insufficient Fe uptake always leads to Fe-deficiency symptoms such as interveinal chlorosis in leaves and a reduction in crop yield (Briat et al., 2015). Peanut is widely cultured in China and is a potential source of Fe (Akram et al., 2018). At the same time, the nitrogenase in nodulation and nitrogen fixation also requires Fe (Briat et al., 2015). However, most peanuts in the world are planted in alkaline calcium areas, resulting in Fe deficiency, chlorosis, photosynthesis obstruction, nutrient accumulation reduction, and serious impact on crop yield (Lingenfelser et al., 2005). Moreover, Fe deficiency in plants has also been proven to cause a strong stress response in plants following excessive H_2_O_2_ and ·${\text{O}}_{2}^{-}$ production, leading to damage to the plant immune system and even cell death (Czarnocka and Karpinski, 2018). Therefore, it is very urgent to find a sustainable and environmentally friendly method to promote Fe absorption in plants.

Due to the serious impact of Fe deficiency on plant growth, plants have evolved two strategies to take up Fe from the soil. Grasses, such as corn, wheat, and rice, use the chelation-based Strategy II. In response to Fe-deficiency, grasses release small molecular compounds known as the mugineic acid (MA) family of phytosiderophores (PS) (Romheld and Marschner, 1986). Dicots and non-graminaceous monocots (non-grass species) employ the reduction strategy, named Strategy I (Kim and Guerinot, 2007). The first step is the acidification of the plant rhizosphere via the activity of specific H^+^-ATPases (Santi and Schmidt, 2009). Then, Fe(III) chelate reductases reduce Fe(III) into Fe(II) that is up taken into the root via transporters of the Zip family (Connolly et al., 2003; Wang et al., 2017), In *Arabidopsis thaliana*, these three steps are, respectively, mediated by AHA2, FRO2, and IRT1. In terms of transcription, Fe acquisition is controlled by a series of regulatory events, of which *FIT* plays a predominant role in sustaining and restricting the amount of Fe in plant roots of eudicots (Yuan et al., 2005). Gene co-expression analysis has defined different Fe-related regulatory modules. The first module relies on the activity of (FER-like iron deficiency induced transcription factor (*FIT/ bHLH29*), a clade IIIa *bHLH* TF (Colangelo and Guerinot, 2004). *FIT*/bHLH29 is a direct regulator of *IRT1* and *FRO2* expression, highlighting its central role in the regulation of the Fe uptake machinery. The second module acts upstream from FIT/bHLH29. It involves the four members of the IVc *bHLH* clade, namely ILR3/bHLH105 (IAA-LEUCINE RESISTANT 3), *IDT1/bHLH34* (IRON DEFICIENCY TOLERANT 1), *bHLH*104, and *bHLH*115. These four TFs play additive roles in response to Fe deficiency and their activity is thought to rely at least in part on their ability to form homo-or heterodimers (Liang et al., 2017; Gao and Dubos, 2021), these four TF also interact with the b*HLH*121 master regulator (Gao et al., 2020; Gao and Dubos, 2021). Functional homologs of most of the above-described Arabidopsis *bHLH* TFs have been characterized in several dicots, indicating that this regulatory mechanism is most likely conserved among Strategy I plants (Gao and Dubos, 2021).

Endophytic fungi can promote plant growth and yield and can act as biocontrol agents by promoting plant growth by producing a range of natural products that can be harnessed for potential use in medicine, agriculture, or industry (Rodriguez et al., 2009). A beneficial endophyte *P. liquidambaris* was isolated from our laboratory in the early stage and has been reported to be symbiotic with peanut (Zhang et al., 2016), rice (Sun et al., 2019), and Arabidopsis (Zhang et al., 2019). In addition, it can promote Fe absorption in peanut (Su et al., 2019). One possible reason is that endophytes regulate the hormone signaling pathway, which in turn changes the plant element absorption ability. However, the detailed mechanism needs to be elucidated. Therefore, the purpose of this study was to clarify the possible mechanism by which *P. liquidambaris* alleviates the Fe deficiency in peanuts.

In our study, an H_2_O_2_ decrease after *P. liquidambaris* colonization under Fe-deficiency in peanuts was reported. It has been reported that H_2_O_2_ plays a negative regulatory effect on Fe absorption in other plants (Ranieri et al., 2003; von der Mark et al., 2021). However, H_2_O_2_ is not only a stress molecule but also an important signaling pathway in plants. A high H_2_O_2_ always means high oxidative stress damage. The role of H_2_O_2_ in the Fe absorption of peanut promoted by *P. liquidambaris* is currently unknown. We designed this study to clarify the role of H_2_O_2_ play in *P. liquidambaris* promoting Fe absorption in peanuts. We hypothesize that H_2_O_2_ participates in *P. liquidambaris*-regulated Fe absorption by regulating the expression of proteins involved in Fe absorption and detecting them.

## Materials and Methods

### Plant Cultivation

Peanut (line Gan hua-5) seeds were obtained from the Jiangxi Ecological Experimental Station of Red Soil, Chinese Academy of Science, surface sterilized (5 min in 70% EtOH; rinse sterile water), germinated in the dark at 28°C with autoclaved damp vermiculite until the radicle reached 2–3 cm and then transferred to 1/2 Hogland nutrient solution [6 mM/L KNO_3_, 5 mM/L CaCl_2_, 1 mM/L NaH_2_PO_4_, 2 mM/L MgSO_4_, 0.025 mM/L H_3_BO_3_, 2 μM/L MnCl_2_, 1 μM/L ZnSO_4_, 0.1 μM/L (NH_4_)_6_Na_7_MoO_24_, 0.25 μM/L CuSO_4_ and 100 μM/L Fe-EDDHA, pH 6.0] until different treatments, and kept in a growth chamber at a constant temperature of 28°C on a day/night cycle of 16/8 h.

### Endophytic Colonization and Assay

The fungal strain *P. liquidambaris* was originally isolated from the inner bark of *Bischofia polycarp* and inoculated on potato dextrose agar, labeled with a green fluorescent protein (GFP) through a vector plasmid pCT74 by Chen et al. (2011). The fungal inoculum was centrifuged before use, and then the mycelium was washed and dissolved with sterilized double-distilled water for inoculation and irrigated to the roots. Total genomic DNA from plant roots was extracted 7 days after inoculation to detect colonization by qPCR, and GFP primers were used (Supplementary Table 1). The fungi treatment was expressed as E in the Figure. For the H_2_O_2_ treatment, different concentrations (20, 40, 80, 100, and 200 μmol/L) were used, for the catalase, CAT-1 (5 mKat/L), and CAT-2 (10 mKat/L) were chosen according to the previous study (Xie et al., 2017).

### Determination of Morphological and Photosynthesis Features

Once harvested, root and shoot weights were measured using a digital scale. The chlorophyll content of young developed leaves was measured by acetone extraction. Accurately, weighed plant samples (0.1 g) were homogenized in the presence of 1 ml 80% acetone and leached for 2 h. Each treatment was performed in six parallel samples. Subsequently, the chlorophyll content of the samples was determined using a spectrophotometer at 663 and 645 nm. The calculation of chlorophyll pigment concentrations was performed according to the following equation (Arnon, 1949):


$$\begin{array}{l} Chl\;Total=\frac{\left[20.2\left(A645\right)+8.2\left(A663\right)\right]\times V}{\left(1000\times W\right)} \end{array}$$

### Fe Concentration Detection With Atomic Absorption Spectroscopy and Perl Staining

Peanut root and shoot samples were dried in an oven at 80°C until a constant weight was achieved. Then, 0.1 g of dried sample was placed in a digestion tube filled with 5 ml of nitric acid and digested using a microwave digestion apparatus. The digested sample was diluted to 50 ml with distilled water, and the Fe content was then determined using an atomic spectrophotometery (Prodigy, Leeman, USA). To observe the localization of Fe in vetiver plants, fresh root tissues were processed and stained with Perls Prussian blue (Stacey et al., 2008). Fresh roots were infused with 4% (v/v) HCl and 4% (w/v) potassium ferrocyanide (Perls Prussian blue stain). Excess stain was washed with distilled water, and the slides were photographed using a digital camera.

### Analysis of Cell Death, H_2_O_2_, and ·${\text{O}}_{2}^{\text{–}}$ in Root and Shoot

Cell death was tested with minor modifications with the Evans blue method (Kabir et al., 2020). Briefly, at room temperature, shoots and roots were initially incubated in 0.25% Evans blue emulsion for ~15 min. The suspension was subsequently treated with 1 ml of 80% ethyl alcohol for 10 min. The plant tissues were next incubated at 50°C for 15 min in a water bath and then rotated at 12,000 rpm for 10 min. The supernatant's optical absorption was finally measured at 600 nm. H_2_O_2_ was determined using a kit purchased from Jiancheng (Nanjing, China).

For the measurement of the ·${\text{O}}_{2}^{-}$ generation rate, 0.3 g of fresh samples were ground in liquid N_2_ and extracted in 3 ml of ice-cold 50 mM sodium phosphate buffer (PBS) (pH 7.0). About 1 ml of fresh leaf extract supernatant was added to 0.9 ml 65 mM phosphate buffer solution (pH 7.8) and 0.1 ml 10 mM hydroxylammonium chloride. PBS (pH 7.0) instead of 1 ml of fresh leaf extract supernatant was used as a blank. The reaction was incubated at 25°C for 35 min. The solution from the above reaction mixture (0.5 g) was then added to 0.5 ml of 17 mM sulfanic acid and 0.5 ml of 7.8 mM α-naphthylamine solution. After 20 min of reaction, 2 ml of ether was added to the above solution and then mixed well. The solution was centrifuged at 1,500 g at 4°C for 5 min. The absorbance of the pink supernatant was measured at 530 nm with a spectrophotometer. Absorbance values were calibrated to a standard curve generated with known concentrations of HNO_2_ (Wang Q. H. et al., 2013).

### RNA Extraction, CDNA Synthesis, and QPCR Analysis

Total RNA was extracted from the root samples using a total RNA isolation reagent (Sangon, Shanghai) following the manufacturer's instructions, and the final RNA yield was quantified using a Nanodrop Spectrophotometer. First-strand cDNA synthesis was performed from RNA with a cDNA synthesis kit (Vazyme, China). Quantitative real-time PCR was performed in a real-time PCR system to detect the expression of Ah*IRT*1, Ah*FIT*, and Ah*FRO2* using specific primers (Supplementary Table 1). The levels of relative gene expression were analyzed using the 2^−ΔΔCt^ method, and the housekeeping gene Ah*Actin* was used as an internal control. Six independent replicates were considered for each sample.

### Analysis of Stress Indictors

Superoxide dismutase (SOD) activity was assayed by measuring its capacity to inhibit the photochemical reduction of nitroblue tetrazolium following the method of Stewart and Bewley (1980). Peroxidase (POD) activity was measured by the increase in absorbance at 470 nm due to guaiacol oxidation (Nickel, 1969). Catalase (CAT) activity was measured as the decline in absorbance at 240 nm due to the decrease in the extinction of H_2_O_2_ according to the method of Patra et al. (1978). The reduced glutathione (GSH) content assays according to the previous study (Li et al., 2013). VC was determined by titration with 2,6-dichlorophenolindophenol (Bessey, 1933). For the nicotinamide adenine dinucleotide phosphate (NADPH) detection, fresh tissues were ground into powder in liquid nitrogen and dissolved in an extraction solution (50 mM Tris-HCl, 0.25 M sucrose, 1 mM ASC, 1 mM EDTA, 0.6 % PVP, 1 mM PMSF), and then the homogenate was centrifuged at 12,000 rpm at 4°C for 20 min. A 20 μl of an aliquot of the supernatant was mixed by adding 0.5 ml of 10 μM NADPH and 50 mM XTT. The level of lipid peroxidation in fresh leaves was measured in terms of MDA con centration by the thiobarbituric acid reaction method (Heath and Packer, 1968). Fresh leaves and roots were homogenized in 0.05 mol/L phosphate buffer (pH 7.8) with a mortar and pestle under chilled conditions with liquid nitrogen. The homogenate was filtered through a four-layer muslin cloth filter and centrifuged at 12,000 g for 10 min at 4°C. To estimate .OH production, the reaction was performed according to the method reported by Gómez-Toribio (Cheeseman et al., 1988). The reaction mixture contained 500 μM quinone, 100 μM Fe^3+^- 110 μM EDTA, 0–25 μM IlLPMO9A, 100–2500 U/L GDH, 5 mM glucose, and 2.8 mM 2-deoxyribose in 100 mM phosphate buffer (pH 5.0). The production of .OH was estimated as the conversion of 2-deoxyribose into thiobarbituric acid (TBA) reactive substances (TBARS). The absorbance of TBARS was determined at 532 nm.

### RNA Sequencing Analysis

Total RNA was extracted from peanut roots 7 days after *P. liquidambaris* cocultivation. Briefly, RNA was quantified and qualified using an Agilent 2100 Bioanalyzer, NanoDrop (Thermo Fisher Scientific, Waltham, MA, USA), and 1% agarose gel. A total of 1 μg of RNA with an RNA integrity number value >7 was used for subsequent library preparation. Next-generation sequencing library preparations were constructed according to the manufacturer's protocol. Transcripts in FASTA format were converted from a known GFF annotation file and indexed properly. The DESeq Bioconductor package was used for differential expression analysis. The *p*-value was set below 0.05 to detect differentially expressed genes. Gene Ontology (GO) TermFinder was used to identify GO terms, and a list of significantly enriched genes (*p* < 0.05) was annotated. All Illumina sequence data have been deposited in Sequence Read Archive with the project ID PRJNA779473 (https://www.ncbi.nlm.nih.gov/).

### Statistical Analysis

All experiments were performed in at least six replicates except RNA-seq (3 replicates). The data were analyzed using R-Studio, the *t*-test was used to analyze differences between two groups, and Duncan was used when more groups existed. Statistical significance was determined using one-way ANOVA. Different letters represent differences between different groups, and significance is defined as *p* < 0.05. Graphs and images were assembled using Adobe Photoshop 7.0.

## Results

### Effect of *P. liquidambari* on Peanut Grown in Calcareous Calcium Soil

**Figure 1:**
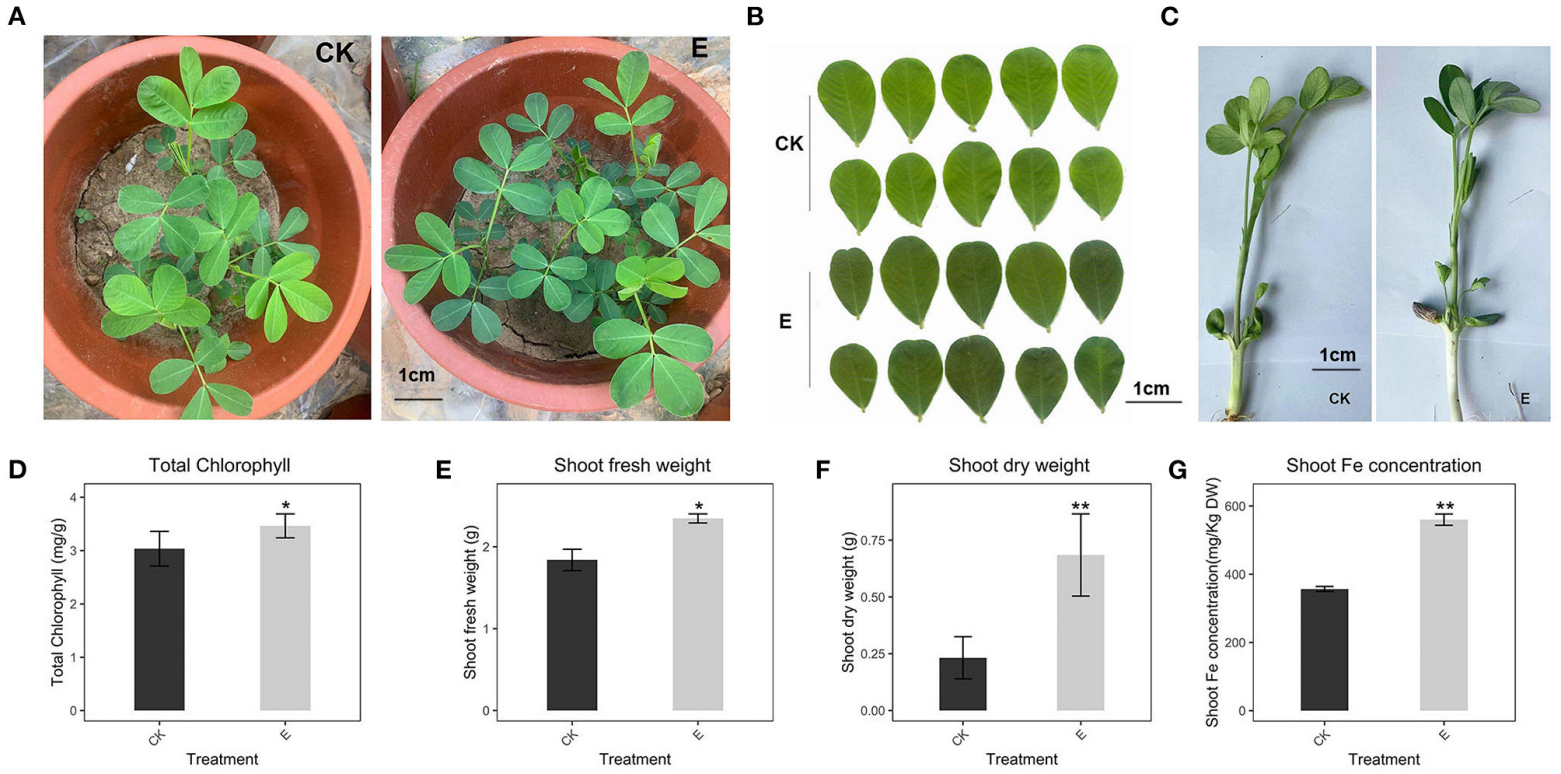
Effect of *P. liquidambaris* on peanut growth in alkaline soil. **(A)**: Effect of *P. liquidambaris* on peanut appearance in pot experimental. **(B)**: Effect of *P. liquidambaris* on peanut leaf color. **(C)**: Effects of *P. liquidambaris* on peanut shoot in seedling stage. **(D)**: Effect of *P. liquidambaris* on chlorophyll content of peanut. **(E)**: Effect of *P. liquidambaris* on shoot fresh weight of peanut. **(F)**: Effect of *P. liquidambaris* on shoot dry weight of peanut. **(G)**: Effect of *P. liquidambaris* on shoot Fe concentration of peanut. Data and errors are mean ± SD (*n* = 6). Black asterisks indicate the significant differences between groups (*p < 0.05; **p < 0.01; *t*-test). E = *P. liquidambaris* inoculation. CK = Alkaline soil without *P. liquidambaris* inoculation.

Plants showed leaf chlorosis in alkaline soil (Figures 1A,B), which seriously affects the chlorophyll synthesis and plant fresh weight (Figures 1D–F). Through the pot experiments (pH 8.0, DTPA-Fe, 3 mg/kg), we learned that in alkaline soil, the addition of *P. liquidambari* significantly promoted a 14% increase in chlorophyll (Figure 1D), a 27.8% increase in fresh weight of the aboveground part (Figure 1E), 50.68% and 81.01% increase in Fe concentration of the root and shoot (Figure 1G and Supplementary Figure 1A), indicating a significant promotion in Fe absorption following *P. liquidambaris* inoculation. At the same time, we also observed changes in plant appearance. *P. liquidambaris* makes the plant greener and grows better in the seedling stage (Figures 1A–C). The Fe concentration, fresh weight, and dry weight of the roots were also improved significantly, and the results were shown in the figure (Supplementary Figures 1A–C). We also compared peanuts grown in alkaline soil (pH 8.0) and acidic soil (pH 6.0) in the early stage. It can be seen from Supplementary Figure 1D that the color of peanut leaves in alkaline soil showed chlorotic, and the chlorophyll is also significantly lower than in acidic soil (pH6.0) (Supplementary Figure 1E). This suggested that peanuts grown in alkaline soils also exhibit chlorophyll synthesis disorders.

### Effects of *P. liquidambaris* on Cell Death, H_2_O_2_, and ·O^2−^

**Figure 2:**
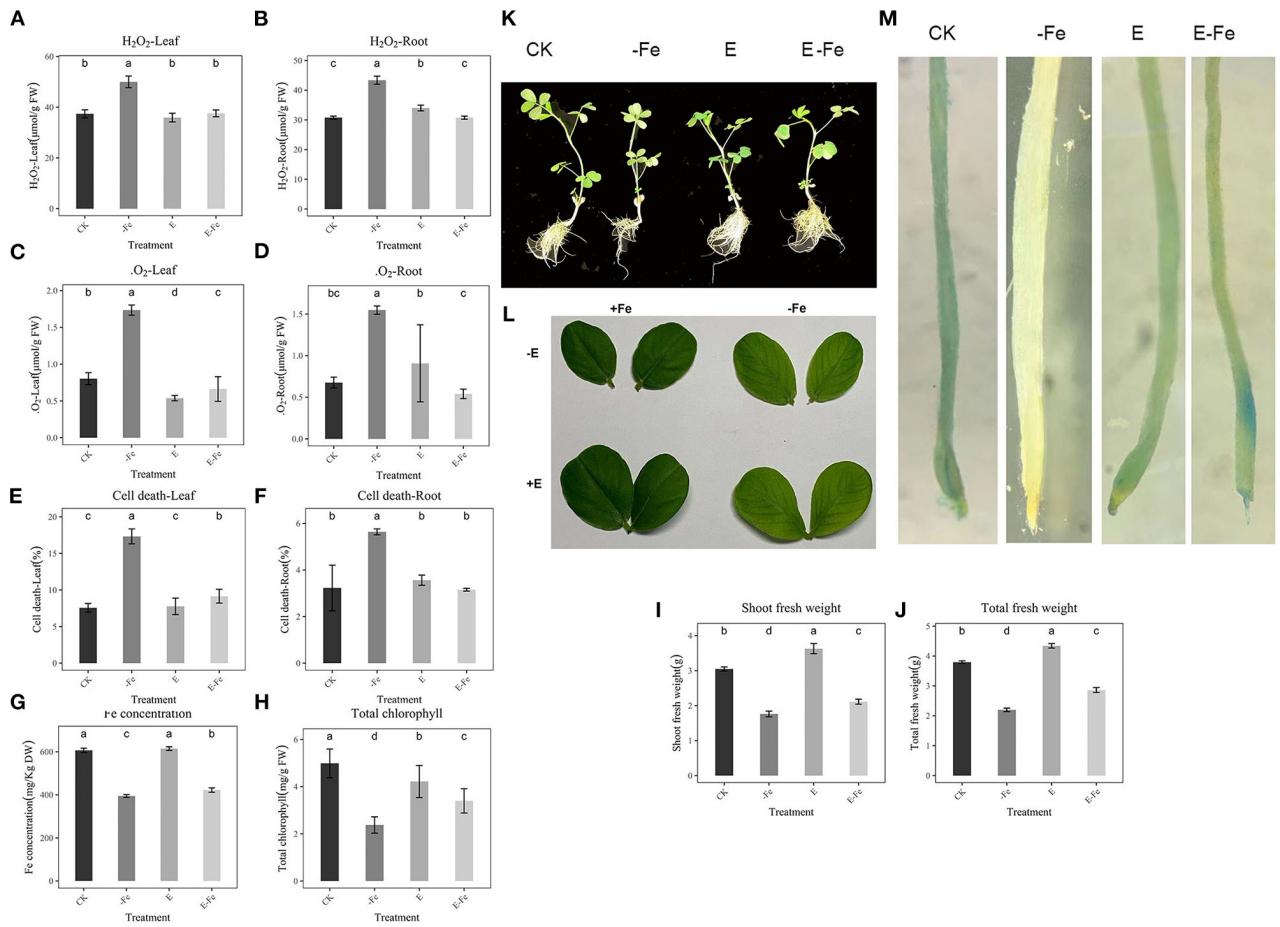
Effect of *P. liquidambaris* and Fe-deficiency on peanut stress indicators. **(A)**: Effect of *P. liquidambaris* and Fe-deficiency on peanut H_2_O_2_ content in leaf. **(B)**: Effect of *P. liquidambaris* and Fe-deficiency on peanut H_2_O_2_ content in root. **(C)**: Effect of *P. liquidambaris* and Fe-deficiency on peanut ·O^2−^ content in leaf. **(D)**: Effect of *P. liquidambaris* and Fe-deficiency on peanut ·O^2−^ content in root. **(E)**: Effect of *P. liquidambaris* and Fe-deficiency on peanut cell death in leaf. **(F)**: Effect of *P. liquidambaris* and Fe-deficiency on peanut cell death in root. **(G)**: Effect of *P. liquidambaris* and Fe-deficiency on peanut Fe concentration. **(H)**: Effect of *P. liquidambaris* and Fe-deficiency on peanut chlorophyll content. **(I)**: Effect of *P. liquidambaris* and Fe-deficiency on peanut shoot fresh weight. **(J)**: Effect of *P. liquidambaris* and Fe-deficiency on peanut total fresh weight. **(K)**: Effect of *P. liquidambaris* and Fe-deficiency on peanut phenotype. **(L)**: Effect of *P. liquidambaris* and Fe-deficiency on peanut leaf. **(M)**: Effect of *P. liquidambaris* and Fe-deficiency on peanut Fe staining in root. Data and errors are mean ± SD, *n* = 6, and different letters indicate significant differences among treatments. *p* < 0.05. E = *P. liquidambaris* inoculation. -Fe = 2 μmol FeEDTA in Hogland nutrition. CK = 100 μmol FeEDTA in Hogland nutrition.

To examine whether *P. liquidambaris* attenuated Fe deficiency-induced damage in peanuts, we analyzed cell death, H_2_O_2_, and ·${\text{O}}_{2}^{-}$. According to the results, Fe deficiency significantly increased H_2_O_2_ (33.7%, 31.8%, Figures 2A,B), ·${\text{O}}_{2}^{-}$ (128%, 30.95% Figures 2C,D), and cell death (129%, 116%, Figures 2E,F) in leaves and roots, indicating serious cell damage. However, the addition of *P. liquidambaris* to Fe-deficient plants showed a significant decrease in these indicators, which were similar to those cultivated under Fe-sufficient conditions. At the same time, the Fe concentration and chlorophyll content are also consistent with the results of Fe staining. The Fe concentration, chlorophyll, and fresh weight of the E-Fe group are significantly higher than that of the -Fe group (Figures 2G–J). The growth of the plant in the seedling stage shows a better growth state, and the leaves show a deeper green (Figures 2K,L). In addition, compared with the CK, Fe staining in roots showed a significantly weak blue color due to Fe deficiency, *P. liquidambaris* supplementation significantly strengthened the blue color of E-Fe plants (Figure 2M) in the root. In Prussian blue dyeing, dark blue represents a higher Fe concentration (Banerjee et al., 2019). The plants showed similar staining levels in roots in the CK and CK+E plants. Compared with the CK group, the Fe concentration has no obvious difference in CK+E, and there is a small change in chlorophyll and fresh weight.

### Effect of Fe Concentration on Fe Absorption Gene Expression

**Figure 3:**
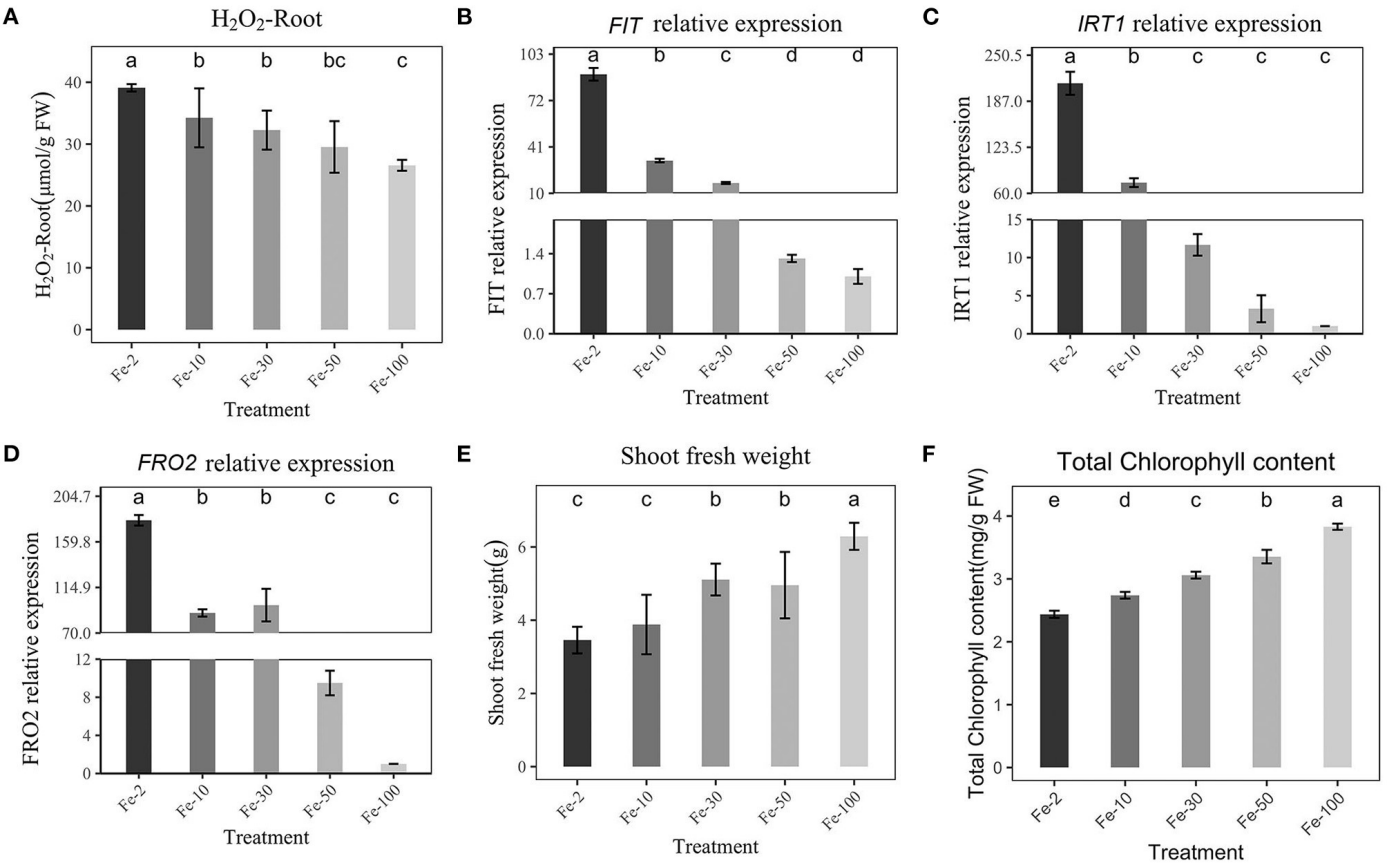
Effects of Hoagland nutrient solution with different Fe concentration on peanut **(A)**: Effects of Hoagland nutrient solution with different Fe concentration on H_2_O_2_ content. **(B)**: Effects of Hoagland nutrient solution with different Fe concentration on *FIT* expression. **(C)**: Effects of Hoagland nutrient solution with different Fe concentration on *IRT*1 expression. **(D)**: Effects of Hoagland nutrient solution with different Fe concentration on *FRO2* expression. **(E)**: Effects of Hoagland nutrient solution with different Fe concentration on fresh weight of shoot. **(F)**: Effects of Hoagland nutrient solution with different Fe concentrations on chlorophyll content. Data and errors are mean ± SD, *n* = 6, and different letters indicate significant differences among treatments. *p* < 0.05. (Fe-2 10, 30, 50, 100, different number means different FeEDTA(μmol) in Hogland nutrition).

To test the correlation between *FIT, IRT*1, *FRO2*, and Fe concentration, we designed experiments with different Fe concentrations and quantified the expression of *FIT, IRT*1, and *FRO2* using RT–qPCR after Fe-deficiency. First, the H_2_O_2_ content in Fe-deficient plants was significantly higher than that in the Fe-sufficient treatment (100 μmol/L, Figure 3A). Then, we found that *FIT* was negatively correlated with Fe concentration. When the Fe concentration was 2 μmol/L, the expression of *FIT* was 89.5 times higher than that at 100 μmol/L (Figure 3B). *IRT*1 and *FRO2* were also strongly induced 202 times and 172 times higher than normal Fe supply (100 μmol/L), respectively (Figures 3C,D). Meanwhile, *IRT*1 and *FRO2* showed similar expression patterns with *FIT*. The fresh weight and chlorophyll of the shoot were also an indicator of Fe concentration. Therefore, chlorophyll and shoot fresh weight are negatively correlated with Fe concentration (Figures 3E,F). Fresh weight of roots, total fresh weight, and phenotypic changes of plants are shown in Supplementary Figure 2 and they were negatively correlated with Fe concentration, too.

### Effect of H_2_O_2_ on Fe Absorption Gene Expression Under Sufficient Fe

**Figure 4:**
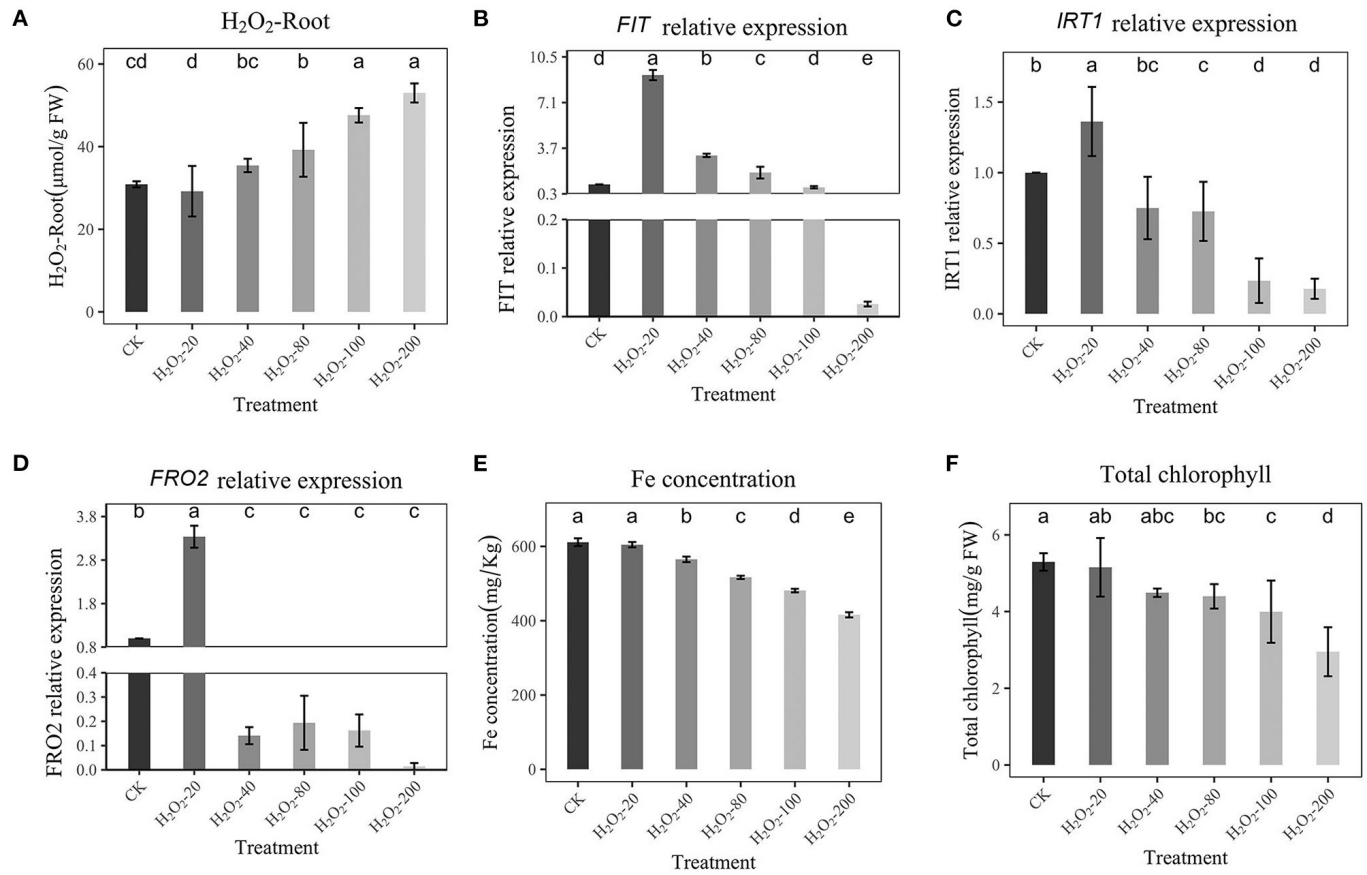
Effects of Hoagland nutrient solution with different H_2_O_2_ concentration on peanut with adequate Fe **(A)**: Effects of Hoagland nutrient solution with different H_2_O_2_ concentration on root H_2_O_2_ content. **(B)**: Effects of Hoagland nutrient solution with different Fe concentration on *FIT* expression. **(C)**: Effects of Hoagland nutrient solution with different H_2_O_2_ concentration on *IRT*1 expression. **(D)**: Effects of Hoagland nutrient solution with different H_2_O_2_ concentration on *FRO2* expression. **(E)**: Effects of Hoagland nutrient solution with different H_2_O_2_ concentration on Fe concentration. **(F)**: Effects of Hoagland nutrient solution with different H_2_O_2_ concentration on chlorophyll content. Data and errors are mean ± SD, *n* = 6, and different letters indicate significant differences among treatments. *p* < 0.05.

To examine the relationship between H_2_O_2_ and Fe absorption, peanuts were treated with different concentrations of H_2_O_2_ (20, 40, 80, 100, and 200 μmol/L) under sufficient Fe supply, and then RT–qPCR was performed to detect the expression levels of *FIT, IRT*1, and *FRO2*. The Fe concentration, H_2_O_2_ content, chlorophyll, and fresh weight of peanut roots were also recorded. The H_2_O_2_ content was shown in Figure 4A. Figures 4B–D shows that a low concentration of H_2_O_2_ had no inhibiting effect on *FIT, IRT*1, *FRO2*, rather, it had a stimulating effect. However, high H_2_O_2_ (80-200 μmol/L) inhibited the expression of *FIT, IRT*1, and *FRO2*, which is consistent with previous experimental results, and the expression of *IRT*1 and *FRO2* was repressed, too (Figures 4A–C). Due to the addition of H_2_O_2_ at a low concentration of (20 μmol/L), the H_2_O_2_ content detected in roots is low (29.19 μmol/g FW), while that under Fe-deficiency is 40.53 μmol/g FW (Figure 4A). Therefore, it may be that the addition of H_2_O_2_ at a low concentration does not increase the H_2_O_2_ content in roots to a high level, so a stimulating effect was observed. However, a high concentration of H_2_O_2_ (80, 100, 200 μmol/L) significantly increased H_2_O_2_ content to 39.19-52.99 μmol/g FW in roots and inhibited the expression of *FIT, IRT*1, and *FRO2* significantly. This also implies that the H_2_O_2_ content in the plant reaches a certain value to inhibit Fe absorption. In addition, the Fe concentration and chlorophyll content were decreased dependent on H_2_O_2_ content (Figures 4E,F). When H_2_O_2_ addition is low, it has no obvious effect on Fe concentration and chlorophyll. Increasing H_2_O_2_ (100 and 200 μmol/L) addition significantly reduced Fe concentration and chlorophyll. This shows that a low external concentration of H_2_O_2_ will not inhibit the absorption of Fe, while a high concentration has an obvious inhibitory effect. The shoot fresh weight and total fresh weight were all significantly reduced (100 and 200 μmol/L, Supplementary Figures 3B,C).

### Effect of H_2_O_2_ on Fe Absorption and Growth With *P. liquidambaris* Under Fe-Deficient

**Figure 5:**
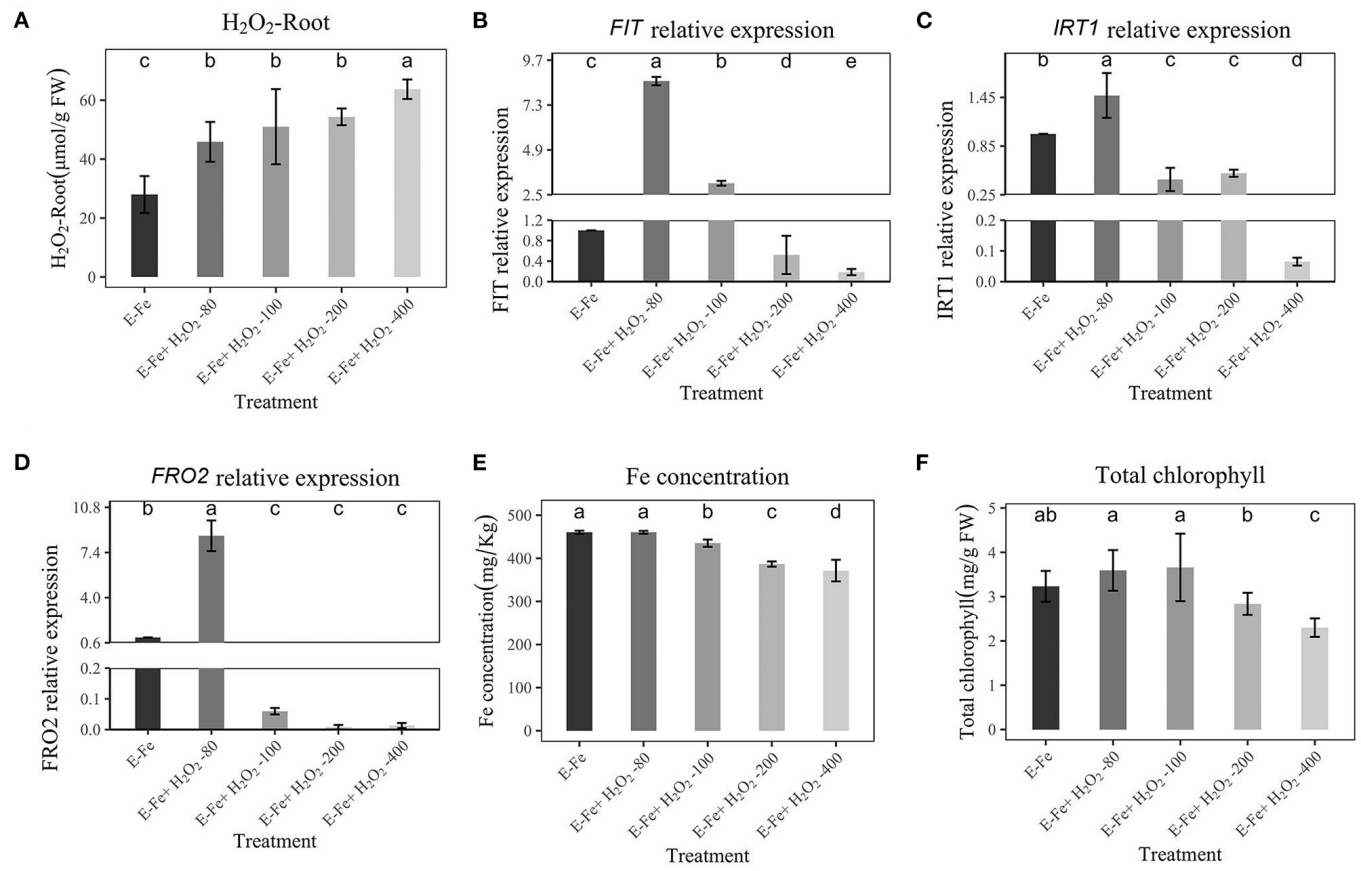
Effects of Hoagland nutrient solution with different H_2_O_2_ concentration on peanut after *P. liquidambaris* colonization under Fe-deficiency **(A)**: Effects of Hoagland nutrient solution with different H_2_O_2_ concentration on H_2_O_2_ content after *P. liquidambaris* colonization under Fe-deficiency. **(B)**: Effects of Hoagland nutrient solution with different H_2_O_2_ concentration on *FIT* expression after *P. liquidambaris* colonization under Fe-deficiency. **(C)**: Effects of Hoagland nutrient solution with different H_2_O_2_ concentration on *IRT*1 expression after *P. liquidambaris* colonization under Fe-deficiency. **(D)**: Effects of Hoagland nutrient solution with different H_2_O_2_ concentration on *FRO2* expression after *P. liquidambaris* colonization under Fe-deficiency. **(E)**: Effects of Hoagland nutrient solution with different H_2_O_2_ concentration on Fe concentration after *P. liquidambaris* colonization under Fe-deficiency. **(F)**: Effects of Hoagland nutrient solution with different H_2_O_2_ concentration on total chlorophyll content after *P. liquidambaris* colonization under Fe-deficiency. Data and errors are mean ± SD, *n* = 6, and different letters indicate significant differences among treatments. *p* < 0.05. E = *P. liquidambaris* inoculation.

Since our results showed that the H_2_O_2_ decreased with the addition of *P. liquidambaris* under Fe deficiency, we explored whether this phenomenon was related to the gene expression of Fe absorption. Considering that *P. liquidambaris* can reduce H_2_O_2_, we increase the concentration of added H_2_O_2_. After peanuts colonized by *P. liquidambaris* were transferred to Fe-deficiency solution, external H_2_O_2_ was added. As shown in Figure 5A, the addition of external H_2_O_2_ (80, 100, 200, 400 μmol/L,) significantly increased H_2_O_2_ in peanut root. When the H_2_O_2_ concentration was artificially changed by external addition, the expression of *FIT, IRT*1, and *FRO2* in peanuts under Fe deficiency also was inhibited, and the inhibition effect was dose-dependent (Figures 5B–D). Furthermore, high *FIT, IRT*1, and *FRO2* expression corresponded to high Fe concentration, and chlorophyll content (Figures 5E,F). Although the expression of *FIT, IRT*1, and *FRO2* was higher when 80 μmol/L H_2_O_2_ than without addition. We have not observed a decrease in Fe concentration and chlorophyll. This may be the same reason as above, that is, low exogenous H_2_O_2_ will stimulate the expression of *FIT, IRT*1, and *FRO2*. The root fresh weight, shoot fresh weight and total fresh weight were all reduced significantly (200 and 400 μmol/L, Supplementary Figures 4B,C), too.

### Effect of CAT on Fe Absorption Under Fe-Deficiency

**Figure 6:**
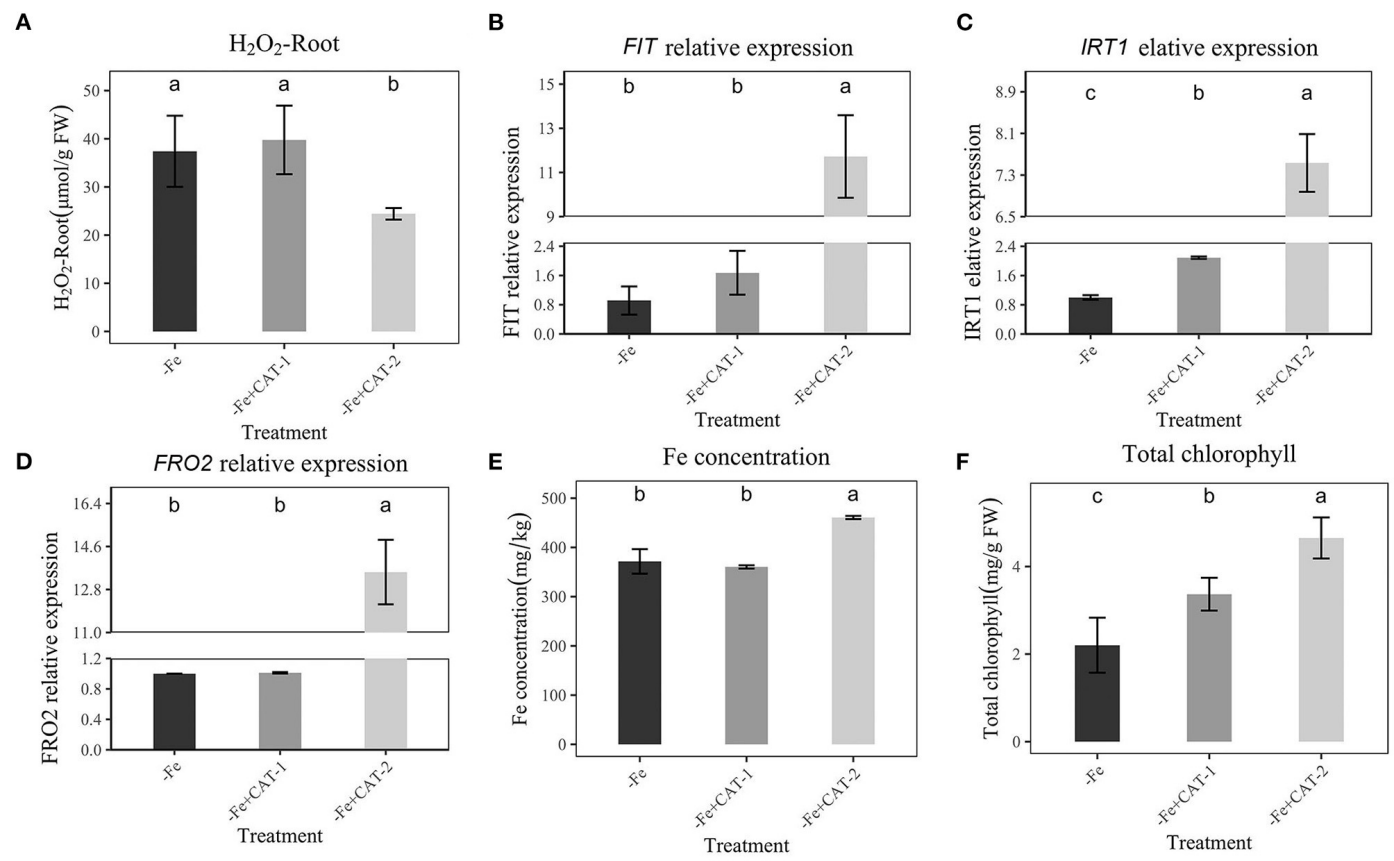
Effects of Hoagland nutrient solution with different CAT concentration on peanut under Fe-deficiency **(A)**: Effects of Hoagland nutrient solution with different CAT concentration on H_2_O_2_ content under Fe-deficiency. **(B)**: Effects of Hoagland nutrient solution with different CAT concentration on *FIT* expression under Fe-deficiency. **(C)**: Effects of Hoagland nutrient solution with different CAT concentration on *IRT*1 expression under Fe-deficiency. **(D)**: Effects of Hoagland nutrient solution with different CAT concentration on *FRO2* expression under Fe-deficiency. **(E)**: Effects of Hoagland nutrient solution with different CAT concentration on Fe concentration under Fe-deficiency. **(F)**: Effects of Hoagland nutrient solution with different CAT concentration on total chlorophyll content under Fe-deficiency. Data and errors are mean ± SD, *n* = 6, and different letters indicate significant differences among treatments. *p* < 0.05. -Fe = 2 μmol FeEDTA in Hogland nutrition. CAT-1 and CAT-2 means two CAT concentrations (5 mKat/L, 10 mKat/L).

To further prove that H_2_O_2_ could affect the expression of the Fe-absorption gene under Fe deficiency, we used CAT to eliminate H_2_O_2_ from plant roots, then performed qRT-PCR to detect the expression of *FIT, IRT*1, and *FRO2*. As shown in Figure 6A, the addition of CAT-2 (10 mKat/L) significantly reduced the H_2_O_2_ content in plant roots. In addition, Fe concentration and chlorophyll under Fe-deficient were increased, along with the high expression of *FIT, IRT*1, and *FRO2* (Figures 6B–D). At the same time, with the addition of 5 mKat/L, we observed no significant change in H_2_O_2_ (Figure 6A) and no change in *FIT* and *FRO2* expression, and a small change in *IRT1* (Figures 6B–D). The Fe concentration and chlorophyll of plants are also consistent with the Fe absorption of plants, 10 mKat/L CAT makes Fe concentration and chlorophyll maximum (Figures 6E,F). At the same time, the shoot fresh weight and total fresh weight were also the largest at this concentration (10 mKat/L, Supplementary Figures 5B,C).

### Effect of *P. liquidambaris* on Stress Indicators Under Fe-Deficiency

**Figure 7:**
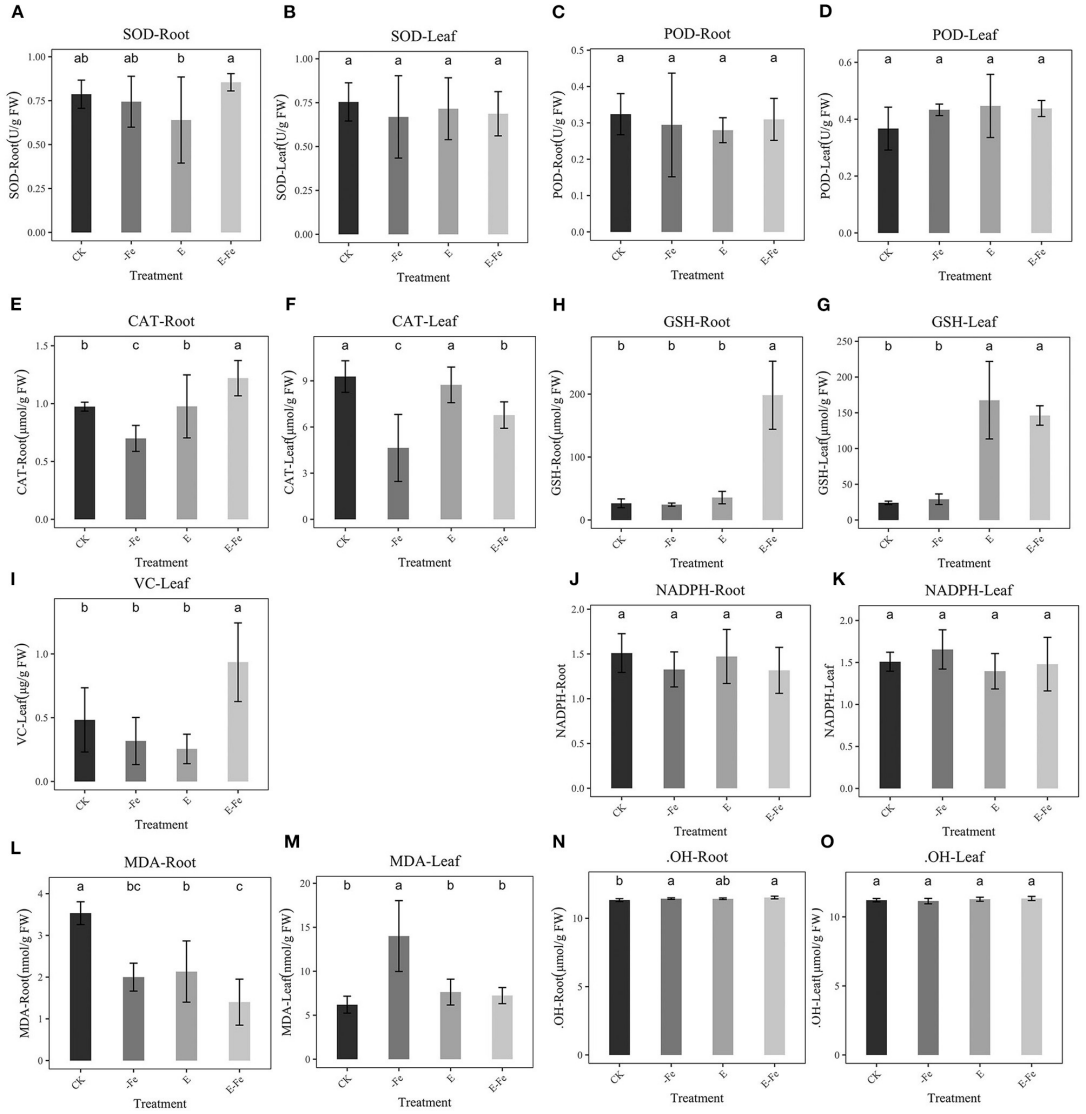
Effect of *P. liquidambaris* on peanut under different Fe supply. **(A)**: Effect of *P. liquidambaris* on peanut SOD content in leaf under different Fe supply. **(B)**: Effect of *P. liquidambaris* on peanut SOD content in root under different Fe supply. **(C)**: Effect of *P. liquidambaris* on peanut POD content in leaf under different Fe supply. **(D)**: Effect of *P. liquidambaris* on peanut POD content in root under different Fe supply. **(E)**: Effect of *P. liquidambaris* on peanut CAT content in leaf under different Fe supply. **(F)**: Effect of *P. liquidambaris* on peanut CAT content in root under different Fe supply. **(G)**: Effect of *P. liquidambaris* on peanut GSH content in leaf under different Fe supply. **(H)**: Effect of *P. liquidambaris* on peanut GSH content in root under different Fe supply. **(I)**: Effect of *P. liquidambaris* on peanut VC in root under different Fe supply. **(J)**: Effect of *P. liquidambaris* on peanut NADPH in root under different Fe supply. **(K)**: Effect of *P. liquidambaris* on peanut NADPH in leaf under different Fe supply. **(L)**: Effect of *P. liquidambaris* on peanut MDA in leaf under different Fe supply. **(M)**: Effect of *P. liquidambaris* on peanut MDA in leaf under different Fe supply. **(N)**: Effect of *P. liquidambaris* on peanut .OH in root under different Fe supply. **(O)**: Effect of *P. liquidambaris* on peanut OH in leaf under different Fe supply. Data and errors are mean ± SD, *n* = 6, and different letters indicate significant differences among treatments. *p* < 0.05. E = *P. liquidambaris* inoculation. -Fe = 2 μmol FeEDTA in Hogland nutrition. CK = 100 μmol FeEDTA in Hogland nutrition.

We detected the contents of different stress indictors to observe whether the addition of *P. liquidambaris* can help peanuts eliminate oxidative damage. SOD in the root was significantly up-regulated in the E-Fe group compared with E, however, it was not significant compared with -Fe. There was no obvious phenomenon in the leaves (Figures 7A,B). No obvious changes were detected in POD (Figures 7C,D). The CAT of roots and leaves decreased significantly in the Fe-deficiency group, while E-Fe increased significantly (Figures 7E,F), however, in the root, the CAT in E-Fe was higher than CK and E while in leaf it's a little lower. The GSH of the E-Fe group increased significantly in leaves and roots compared to the -Fe group (Figures 7G,H), but we did not detect a significant change between the -Fe group and CK. Finally, we observed a significant increase in VC content in the E-Fe group compared to other groups (Figure 7I). But VC can only be detected at the leaves. No obvious changes were detected in NADPH (Figures 7J,K). Compared with CK, the root MDA in E-Fe group decreased significantly, but compared with the -Fe group, the change was not obvious (Figures 7L,M). The MDA of the Fe-deficiency group of the leaf was significantly higher than that of the other groups. No obvious changes were detected in .OH (Figures 7N,O).

### Transcriptome Data Analysis

**Figure 8:**
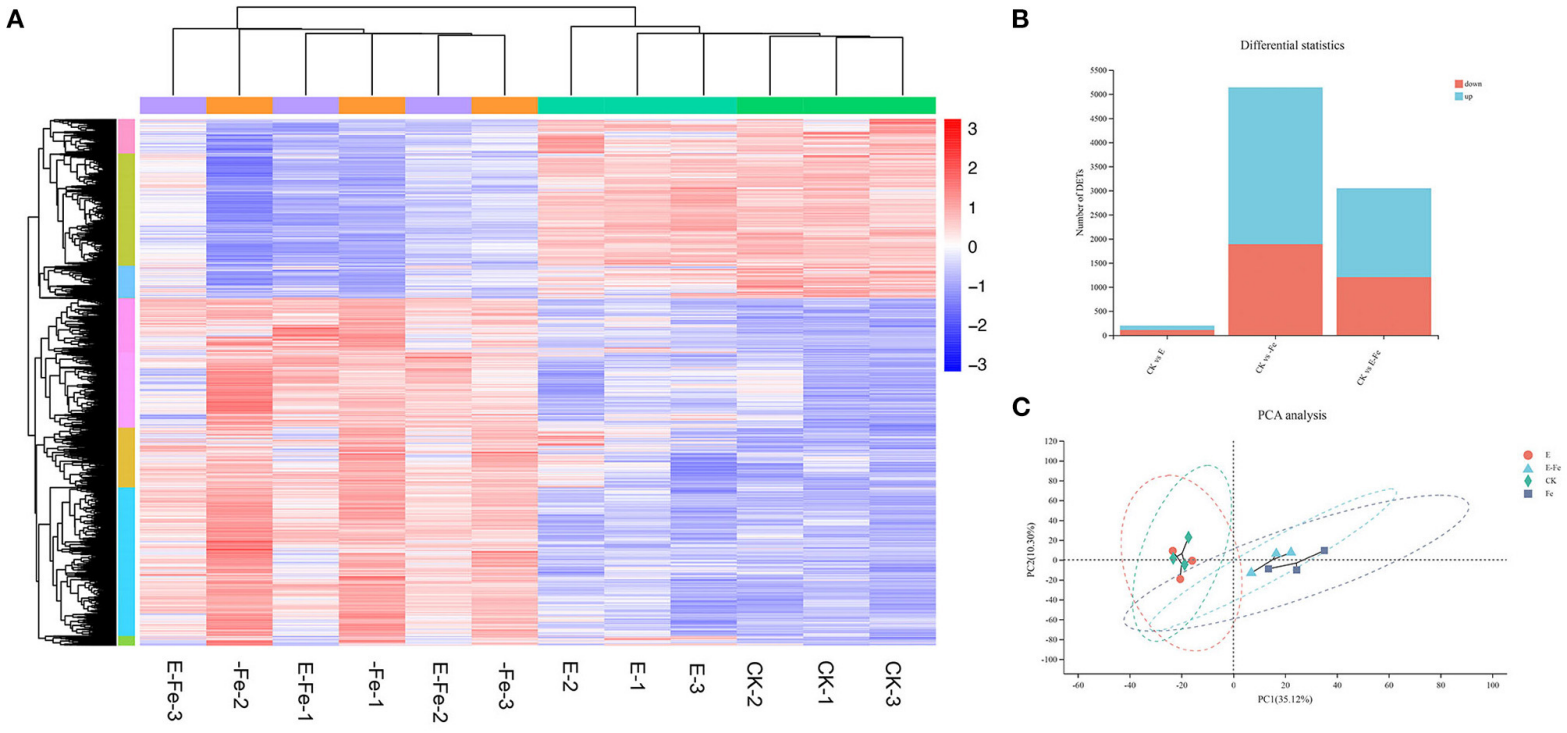
Global analysis of peanut gene expression during early interactions with *P. liquidambaris*. RNA was extracted from peanut root at 7 days after growth on solution with or without *P. liquidambaris* cocultivation, and then RNA-sequencing was performed. **(A)** Differentially expressed genes were hierarchically clustered according to potentially similar functions. **(B)** The Venn diagram depicting the common DEGs. **(C)**: PCoA analysis. E = *P. liquidambaris* inoculation. -Fe = 2 μmol FeEDTA in Hogland nutrition. CK = 100 μmol FeEDTA in Hogland nutrition. Red color represents the high expression of the gene, whereas blue color represents low expression.

After 7 days, post *P. liquidambaris* addition, we used q-PCR to detect the colonization of *P. liquidambaris*. As shown in Supplementary Figure 6, *P. liquidambaris* can be detected in peanut root, it indicated that *P. liquidambaris* successfully symbiosis with peanuts. Then to verify our above experimental results, we performed transcriptomic analysis. Consistent with the above experiment, the time point we selected was 7 days after Fe-deficiency, that is, the 14^th^ day of *P. liquidambaris* colonization. These genes were hierarchically clustered according to similar functions (Figure 8A). According to the Venn diagram (Figure 8B), the addition of *P. liquidambaris* upregulated 79 genes and downregulated 96 genes compared with the CK group, while Fe-deficiency treatment upregulated 3886 genes and downregulated 2008 genes. In the presence of *P. liquidambaris*, Fe deficiency upregulated 62 genes and downregulated 424 genes compared with Fe-deficiency. The early colonization of *P. liquidambaris* reduced the number of different expression genes number in plants caused by Fe deficiency (Figure 8C). For the GO analysis (Supplementary Figures 7A–C), it was found that the phenylpropanolamine metabolic process was the most significantly enriched in the Fe-deficiency group relative to the CK group, followed by glutathione metabolism and metal iron ion metabolism, and secondary metabolism and phenylpropanolamine synthesis were also significantly enriched. When *P. liquidambaris*-colonized peanut was exposed to Fe deficiency, significant enrichment was observed in the regulation process of transcription factors, DNA binding transcription factor activity and specific DNA sequence binding activity. In the *P. liquidambaris* group compared with CK treatment, the cell junctions were significantly enriched (Supplementary Figure 7C). The KEGG results showed that compared with the CK group (Supplementary Figure 7D), the main enrichment pathways in the Fe-deficiency group were flavonoid synthesis, genes regulating plant circadian rhythm, and plant–pathogen interactions. At the same time, the protein process in the endoplasmic reticulum and the MAPK signaling pathway in plants were also significantly activated. In the *P. liquidambaris* treatment group compared with CK peanut (Supplementary Figure 7E), the most significantly enriched signaling pathway was the plant rhythm regulation signaling pathway, followed by the plant–pathogen interaction and RNA degradation pathways, and obvious changes were also observed in ribosome biosynthesis and ion transport. We also found significant changes in the process of photosynthesis. Under Fe deficiency stress after the addition of *P. liquidambaris* with Fe-deficiency (Supplementary Figure 7F), significant changes were observed in phenolic acid synthesis, plant circadian rhythm regulation, plant–pathogen interaction, and phenolic acid biosynthesis. The transport of metal ions, protein synthesis of the endoplasmic reticulum, and MAPK signaling were also activated.

To verify the DEGs identified in RNA-Seq, 8 DEGs were selected for qRT-PCR. qRT-PCR results were compared with the FOLD change of FPKM in RNA-sequencing expression analysis (Supplementary Figure 8). As shown in Supplementary Figure 8, 6 genes showed similar expression patterns in qRT-PCR analysis to RNA-Seq analysis. These results confirm the reliability and accuracy of our transcriptome data.

## Discussion

As Fe plays a pivotal role in photosynthesis, Fe deficiency always affects crop yield and quality. Approximately one-third of cultivated land worldwide belongs to calcareous soils where Fe availability is low. Thus, it is very important to explore and develop an amicable Fe absorption strategy. Endophytic fungi, as beneficial microorganisms of plants, have attracted increasing attention to promoting host nutrition absorption (Chen et al., 2021; Verma et al., 2021). Studies have also reported that endophytic help plants alleviate external abiotic stress (Yan et al., 2019), but the mechanisms remain unclear. In this study, we reported that *P. liquidambaris* assist peanuts in alleviating Fe deficiency stress and explored the possible reasons. Finally, we found that the increase in H_2_O_2_ under Fe deficiency inhibited the expression of *FIT*, which in turn repressed the expression of *IRT*1 and *FRO2* downstream and decreased Fe absorption. The colonization of *P. liquidambaris* reduced the H_2_O_2_ level in peanuts, thus relieving the inhibition of *FIT* by H_2_O_2_. Then the recovery of *IRT*1 and *FRO2* restored the Fe absorption of peanut. The increase in Fe concentration helped peanuts survive Fe deficiency stress and reduced the oxidative stress damage with increases in the CAT, GSH, and MDA.

### *P. liquidambaris* Maintained the Low H_2_O_2_ Level of Peanut Under Fe Deficiency

Reactive oxygen species (ROS) signaling is the key determinant of the plant's environmental response (He et al., 2018). H_2_O_2_ has been shown to negatively regulate Fe absorption in many plants. It can increase in amount under stress and damage proteins, nucleic acids, and lipids, eventually triggering cell death (Ranieri et al., 2003; von der Mark et al., 2021). H_2_O_2_ is not only an oxidative damage substance *in vivo*, but also an important hormone signal (Van Leene et al., 2016). Our results show that the addition of *P. liquidambaris* significantly reduces the H_2_O_2_ of plants under Fe-deficiency, suggesting that Fe-deficiency stress is alleviated. A low H_2_O_2_ content can maintain the normal growth state of plants. Some studies have confirmed that the reduction of H_2_O_2_ content depends on the activation of cat2 as demonstrated in an Arabidopsis cat2 mutant (von der Mark et al., 2021), indicating that cat2 can help metabolize excess H_2_O_2_ in plants. The CAT enzyme activity was higher than that of Fe-deficiency (Figure 7) after *P. liquidambaris* addition. In addition, *IRT*1 functions as a transporter that helps *CAT2* transport Fe, as the activity of *CAT2* requires the participation of Fe. Fe-deficiency will prompt endogenous H_2_O_2_ to produce .OH in the Fenton reaction. This is also the reason why Fe-deficiency causes oxidative damage.

### H_2_O_2_ Negative Regulates Fe Absorption in Peanut

A negative correlation between ROS and Fe acquisition, particularly H_2_O_2_, has been reported (Ranieri et al., 2003), and their relationship has previously been shown to affect *FIT* activity through interaction with the zinc finger, an oxidative stress response transcription factor of Arabidopsis thaliana At*ZAT12* (Brumbarova et al., 2016), thereby negatively regulating Fe absorption. Many studies have reported the relationship between *FIT* and *IRT*1 and *FRO2* (Seguela et al., 2008; Yuan et al., 2008; Maurer et al., 2011; Sivitz et al., 2011; Nishida et al., 2012; Wang N. et al., 2013; Matsuoka et al., 2014). It is agreed that *FIT* is upstream of *IRT*1 and *FRO2* in the regulation of Fe absorption. Even under Fe-deficiency induced by transitional Ni, the overexpression of *FIT, IRT*1, and *FRO1* has been detected (Nishida et al., 2012). Our results also show that *IRT*1 and *FRO2* are consistent with *FIT* under Fe-deficiency, which also supports our speculation. As the downstream result of H_2_O_2_ regulation, *FIT* further regulates the Fe absorption in plants. Although their expression fold changes are not the same. This may be because the low *FIT* can induce high expression of *IRT*1 and *FRO2* or because posttranscriptional modification occurs (Schwarz et al., 2020). It has been reported that phosphorylation of *FIT* regulates the expression of *IRT*1 and *FRO2*, thereby regulating Fe absorption. Some studies suggest that *FIT* may exist in two forms, an active form and an inactive form (Wang et al., 2007). A number of studies have demonstrated that in most plants, *FIT* acts as the upstream of *IRT1* and *FRO2* to regulate Fe uptake in plants (Ling et al., 2002; Ogo et al., 2007; Kim et al., 2019). Although we did not observe the direct effect of *FIT* and *IRT*1 or *FRO2* in peanuts, our qPCR results demonstrated the consistency of their expression, which is consistent with studies in other dicotyledonous plants (Seguela et al., 2008; Yuan et al., 2008; Maurer et al., 2011; Sivitz et al., 2011; Nishida et al., 2012; Wang N. et al., 2013; Matsuoka et al., 2014), we believe that the same regulation pattern also exists in peanut, that is, *FIT* as a transcription factor to regulate the expression of *IRT*1 and *FRO2* and participates in Fe absorption in peanut. At the same time, our experimental results also showed the strong inhibitory effect of H_2_O_2_ on *FIT, IRT*1, and *FRO2*.

### *P. liquidambaris* Protected Peanuts From Oxidative Damage and Promote Peanut Growth

Plants exposed to various biotic and abiotic stresses are compelled to generate higher levels of ROS, such as .OH, ·${\text{O}}_{2}^{-}$ leading to an alteration in the cellular redox homeostasis, therefore, acquiring resistance to neutralizing the excessive oxidative damage (Jin et al., 2021). In the present study, we found that Fe deficiency stress-induced the generation of ·${\text{O}}_{2}^{-}$ and H_2_O_2_ in the leaves of the plants (Figures 2B,C). These results agreed with previous reports by Ranieri et al. (2001) for sunflower and Sun (Sun et al., 2007) for maize. The onset of oxidative damage in plants is more prominent due to Fe deficiency as Fe is the central constituent or factor of major antioxidant enzymes (Kabir et al., 2015). Plants generate ROS when facing the outside stress itself and antioxidant enzymes to resist the damage of the outside world, plant stress relief is usually dependent on the antioxidant enzymes *in vivo*, when Fe deficiency plants showed severe oxidative stress reaction, including H_2_O_2_, ·O^2−^, cell death increased significantly, it is usually harmful to the growth of plants. Our results show that the addition of *P. liquidambaris* can help plants relieve this oxidative stress and tend to a normal growth state, which may be attributed to the increase of antioxidant enzymes in plants. Under the condition of Fe deficiency, the levels of ROS scavenge enzymes (CAT, SOD, POX) of onion seedlings were reported to decrease. CAT could decompose H_2_O_2_ into H_2_O and O_2_ *in vivo*. CAT is an enzyme containing heme, so their activity is correlated with Fe concentration (Sevilla et al., 1984). The elevated Fe concentration enhanced the enzymatic activity of CAT, helping the plants to scavenge more ROS. Although our experimental results did not observe a significant increase in SOD and POD, this may be due to the inappropriate timing of our detection. Transcriptome data also demonstrated that the addition of *P. liquidambaris* helped the plants to far shrink the gene transcriptional changes in the absence of Fe, helping the plants to approach normal growth.

## Conclusion

**Figure 9:**
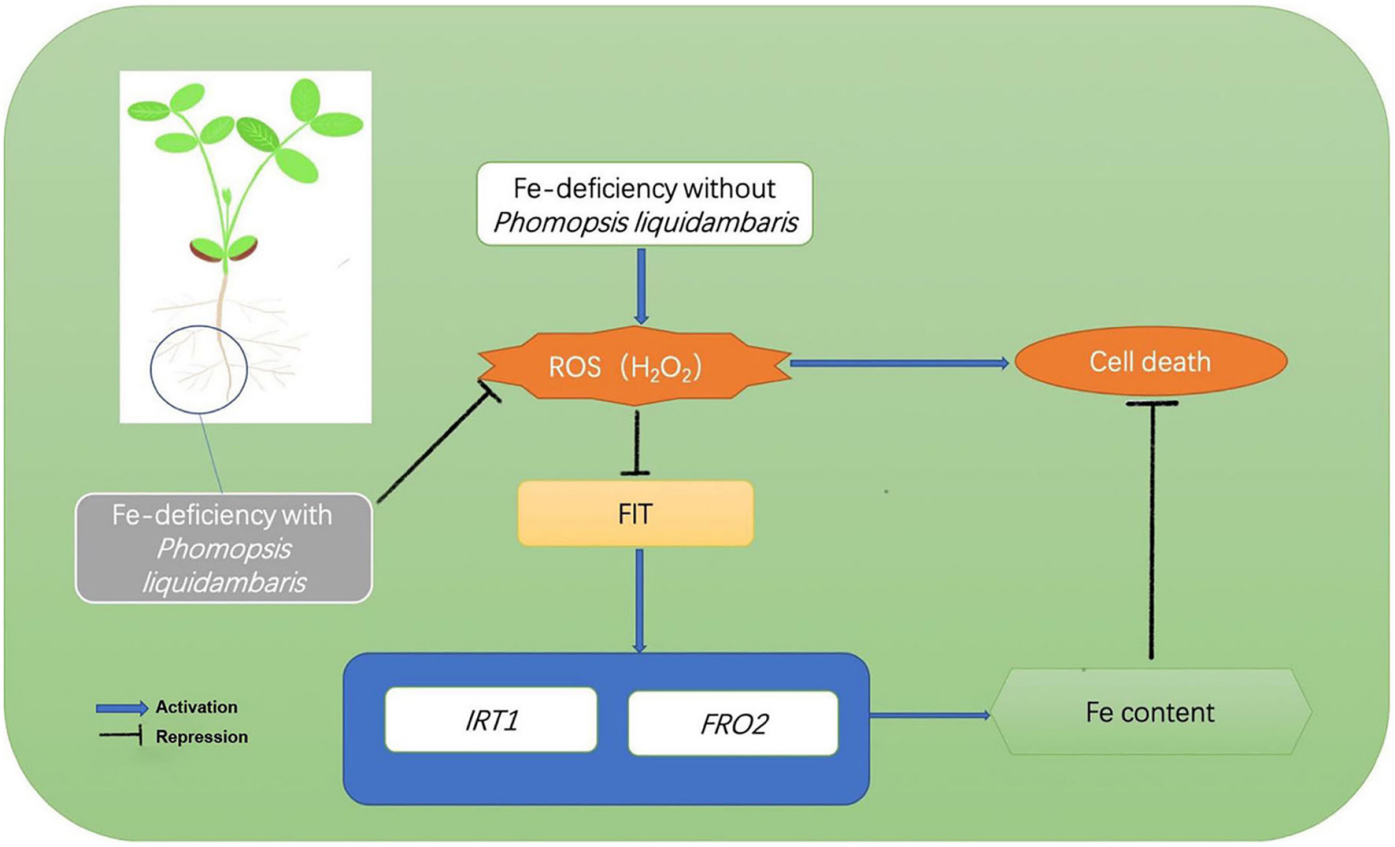
Model of H_2_O_2_-mediated inhibition of Fe acquisition. Under Fe deficiency, H_2_O_2_ was upregulated. H_2_O_2_ represses the transcription of *FIT*, consequently reducing the expression of Fe uptake genes *IRT*1 and *FRO2* and decreasing Fe acquisition from the environment. The colonization of *P. liquidambaris* help plants reduce the H_2_O_2_ content *in vivo*, thereby releasing the *FIT* gene inhibited by H_2_O_2_ and up-regulating downstream *IRT*1 and *FRO2*, then promoting the Fe absorption, and the increased Fe absorption alleviating the Fe-deficiency stress of plants.

Our study supported a model of beneficial plant-microbial interactions under Fe-deficiency, and the endophytic fungi-peanut interaction system demonstrated the potential role of endophytes in improving Fe uptake and thereby alleviating Fe-deficiency stress in plants (Figure 9). That is, under Fe-deficiency, the H_2_O_2_ burst will inhibit the expression of genes related to Fe absorption including *FIT, IRT*1, and *FRO2*, thereby repressing Fe absorption. The addition of *P. liquidambaris* helps plants reduce the synthesis of H_2_O_2_ and eliminate H_2_O_2_, thereby reducing the H_2_O_2_ in plant roots and maintaining H_2_O_2_. The low H_2_O_2_ relieves the limitation of Fe absorption, increases the Fe content, and relieves the Fe deficiency of plants. This will provide theoretical guidance for the promotion and application of beneficial microorganisms in agriculture.

## Data Availability Statement

The datasets presented in this study can be found in online repositories. The names of the repository/repositories and accession number(s) can be found at: National Center for Biotechnology Information (NCBI) BioProject database under accession number PRJNA779473.

## Author Contributions

Material preparation and data collection were performed by Y-CD, L-JK, C-YM, and L-SC. Data analysis was performed by WZ, QZ, and KS. The first draft of the manuscript was written by Y-CD and C-CD. All authors contributed to the article and approved the submitted version.

## Funding

We acknowledge the National Natural Science Foundation of China (Grant No. 31870478), Program for Jiangsu Excellent Scientific and Technological Innovation team (17CXTD00014), and a project funded by the Priority Academic Program Development (PAPD) of the Jiangsu Higher Education Institutions of China.

## Conflict of Interest

The authors declare that the research was conducted in the absence of any commercial or financial relationships that could be construed as a potential conflict of interest.

## Publisher's Note

All claims expressed in this article are solely those of the authors and do not necessarily represent those of their affiliated organizations, or those of the publisher, the editors and the reviewers. Any product that may be evaluated in this article, or claim that may be made by its manufacturer, is not guaranteed or endorsed by the publisher.

## Supplementary Material

Supplementary Figure 1 Effect of *P. liquidambaris* on peanut in alkaline soil. **(A)**: Effect of *P. liquidambaris* on root Fe concentration of peanut. **(B)**: Effect of *P. liquidambaris* on root fresh weight of peanut. **(C)**: Effect of *P. liquidambaris* on root dry weight of peanut. **(D)**: Effects of Different pH soils on peanut leaf color. **(E)**: Effects of Different pH soils on peanut Chlorophyll. Data and errors are mean ± SD, *n* = 6, Black asterisks indicate the significant differences between groups (^*^*p* < 0.05; ^**^*p* < 0.01; *t*-test). E = *P. liquidambaris* inoculation. CK = Alkaline soil without *P. liquidambaris* inoculation.

Supplementary Figure 2 Effects of Hoagland nutrient solution with different Fe concentration on peanut. **(A)**: Effects of Hoagland nutrient solution with different Fe concentration on root fresh weight. **(B)**: Effects of Hoagland nutrient solution with different Fe concentration on total fresh weight. **(C)**: Effects of Hoagland nutrient solution with different Fe concentration on peanut growth. Data and errors are mean ± SD, *n* = 6, and different letters indicate significant differences among treatments. *p* < 0.05 [Fe-2 10, 30, 50, 100, different number means different FeEDTA (μmol) in Hogland nutrition].

Supplementary Figure 3 Effects of Hoagland nutrient solution with different H_2_O_2_ concentrations on peanut with adequate Fe **(A)**: Effects of Hoagland nutrient with different H_2_O_2_ concentrations on root fresh weight. **(B)**: Effects of Hoagland nutrient with different H_2_O_2_ concentrations on shoot fresh weight. **(C)**: Effects of Hoagland nutrient with different H_2_O_2_ concentrations on total fresh weight. Data and errors are mean ± SD, *n* = 6, and different letters indicate significant differences among treatments. *p* < 0.05.

Supplementary Figure 4 Effects of Hoagland nutrient solution with different H_2_O_2_ concentration on peanut after *P. liquidambaris* colonization under Fe-deficiency. **(A)**: Effects of Hoagland nutrient with different H_2_O_2_ concentration on root fresh weight. **(B)**: Effects of Hoagland nutrient with different H_2_O_2_ concentration on shoot fresh weight. **(C)**: Effects of Hoagland nutrient with different H_2_O_2_ concentration on total fresh weight. Data and errors are mean ± SD, *n* = 6, and different letters indicate significant differences among treatments. *p* < 0.05. E = *P. liquidambaris* inoculation. -Fe = 2 μmol FeEDTA in Hogland nutrition.

Supplementary Figure 5 Effects of Hoagland nutrient solution with different CAT on peanut under Fe-deficiency. **(A)**: Effects of Hoagland nutrient with different H_2_O_2_ concentrations on root fresh weight. **(B)**: Effects of Hoagland nutrient with different H_2_O_2_ concentrations on shoot fresh weight. **(C)**: Effects of Hoagland nutrient with different H_2_O_2_ concentrations on total fresh weight. Data and errors are mean ± SD, *n* = 6, and different letters indicate significant differences among treatments. *p* < 0.05. -Fe = 2 μmol FeEDTA in Hogland nutrition.

Supplementary Figure 6 Colonization of *P. liquidambaris* in different treatment groups. The peanuts were transferred to the Hoagland nutrient solution. Seven days later, the colonization was detected by q-PCR. The data shown are the mean ± SD. E =*P. liquidambaris inoculation*. -Fe = 2 μmol FeEDTA in Hogland nutrition. CK = 100 μmol FeEDTA in Hogland nutrition.

Supplementary Figure 7 GO and KEGG pathway enrichment analysis based on the differentially expressed genes between Fe-sufficient and Fe-deficient peanut roots with or without *P. liquidambaris*. **(A)**: Go analysis between CK and -Fe. **(B)**: Go analysis between CK and E. **(C)**: Go analysis between -Fe and E-Fe. **(D)**: KEGG analysis between CK and -Fe. **(E)**: KEGG analysis between CK and E. **(F)**: KEGG analysis between -Fe and E-Fe. E = *P. liquidambaris* inoculation. -Fe = 2 μmol FeEDTA in Hogland nutrition. CK = 100 μmol FeEDTA in Hogland nutrition.

Supplementary Figure 8 The qRT-PCR analysis of genes in the roots under Fe-sufficient or Fe-deficient conditions after *P. liquidambaris* colonization. The relative expression of each gene was calculated as the 2^−ΔΔCT^ value and normalized to the endogenous reference genes. Data and errors are mean ± SD, *n* = 6, and different letters indicate significant differences among treatments. *p* < 0.05. -Fe = 2 μmol FeEDTA in Hogland nutrition. CK = 100 μmol FeEDTA in Hogland nutrition.

Supplementary Table 1 Primers used for RT-qPCR analysis.
