# Supplementary figures and images for: Endophytic Fungus Phomopsis liquidambaris Enhances Fe Absorption in Peanuts by Reducing Hydrogen Peroxide
Source: Front Plant Sci. 2022 Apr 29;13:872242. doi: 10.3389/fpls.2022.872242 (PMC9100952; doi:10.3389/fpls.2022.872242)

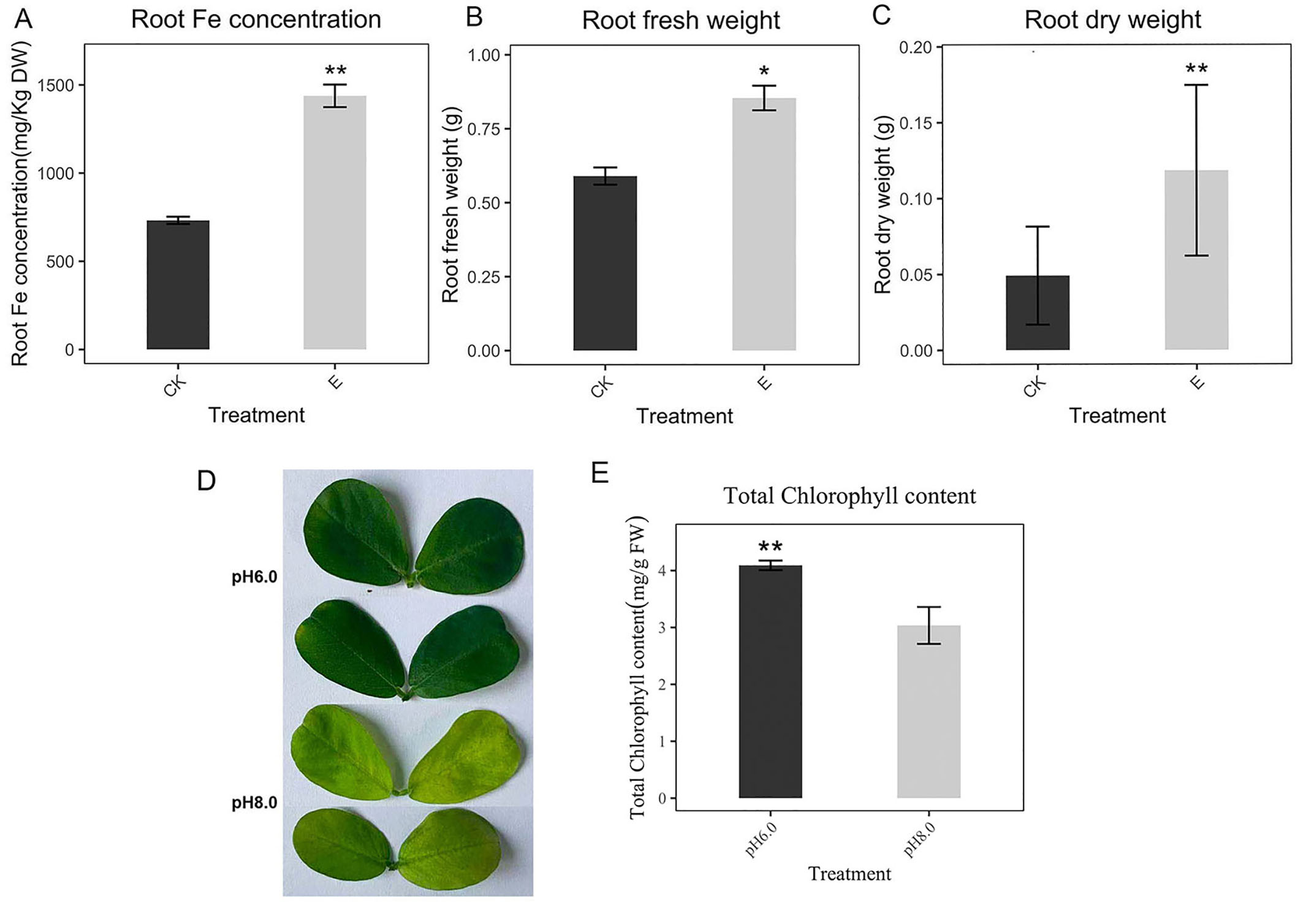

Supplement: Supplementary Figure 1 — Effect of P. liquidambaris on peanut in alkaline soil. (A): Effect of P. liquidambaris on root Fe concentration of peanut. (B): Effect of P. liquidambaris on root fresh weight of peanut. (C): Effect of P. liquidambaris on root dry weight of peanut. (D): Effects of Different pH soils on peanut leaf color. (E): Effects of Different pH soils on peanut Chlorophyll. Data and errors are mean ± SD, n = 6, Black asterisks indicate the significant differences between groups (*p < 0.05; **p < 0.01; t-test). E = P. liquidambaris inoculation. CK = Alkaline soil without P. liquidambaris inoculation. [file Image_1.JPEG]

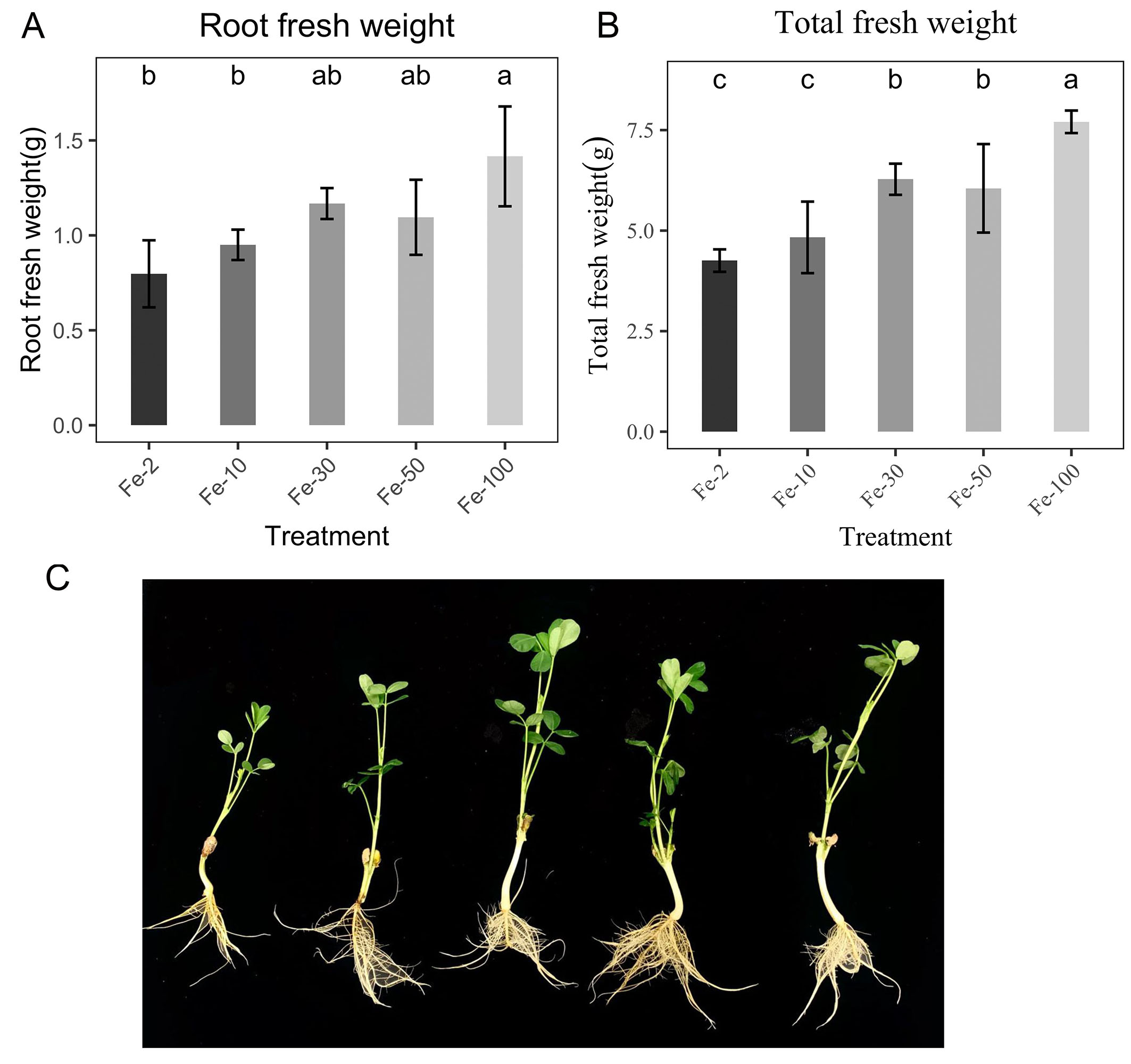

Supplement: Supplementary Figure 2 — Effects of Hoagland nutrient solution with different Fe concentration on peanut. (A): Effects of Hoagland nutrient solution with different Fe concentration on root fresh weight. (B): Effects of Hoagland nutrient solution with different Fe concentration on total fresh weight. (C): Effects of Hoagland nutrient solution with different Fe concentration on peanut growth. Data and errors are mean ± SD, n = 6, and different letters indicate significant differences among treatments. p < 0.05 [Fe-2 10, 30, 50, 100, different number means different FeEDTA (μmol) in Hogland nutrition]. [file Image_2.JPEG]

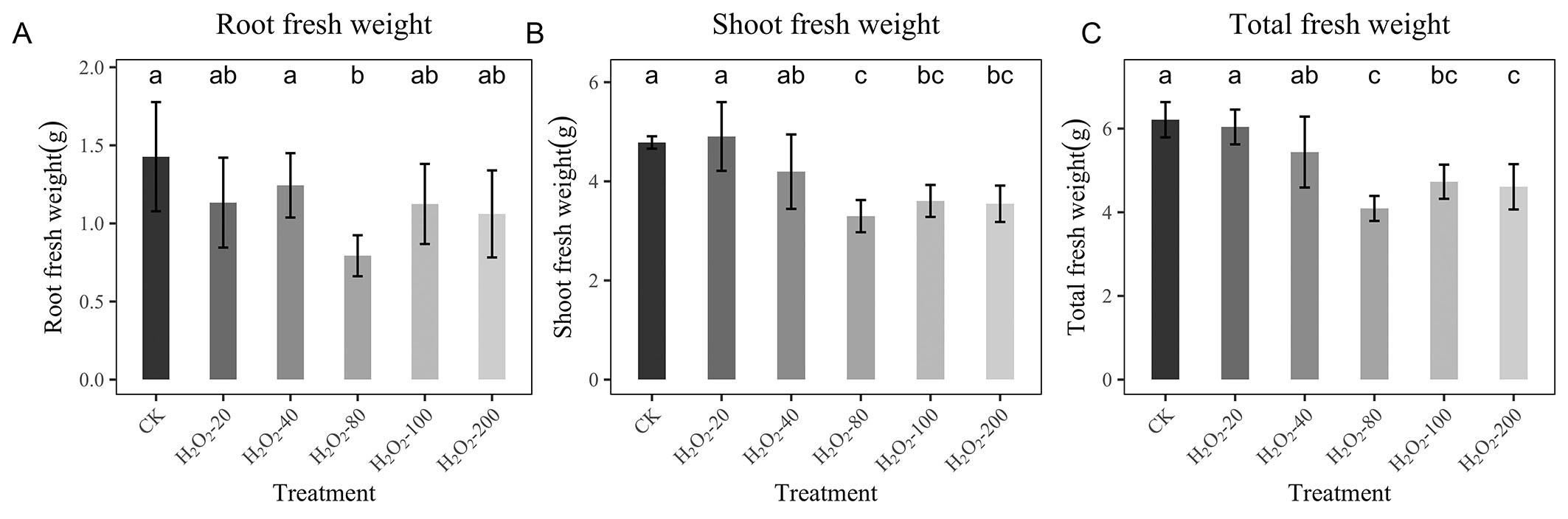

Supplement: Supplementary Figure 3 — Effects of Hoagland nutrient solution with different H2O2 concentrations on peanut with adequate Fe (A): Effects of Hoagland nutrient with different H2O2 concentrations on root fresh weight. (B): Effects of Hoagland nutrient with different H2O2 concentrations on shoot fresh weight. (C): Effects of Hoagland nutrient with different H2O2 concentrations on total fresh weight. Data and errors are mean ± SD, n = 6, and different letters indicate significant differences among treatments. p < 0.05. [file Image_3.JPEG]

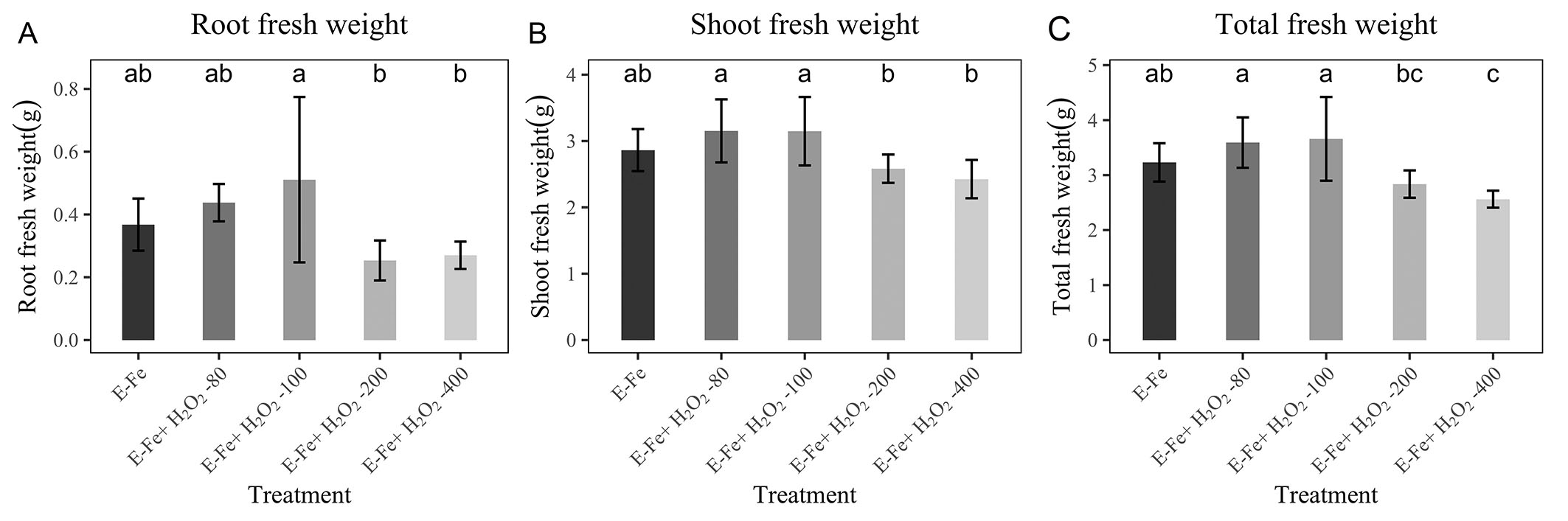

Supplement: Supplementary Figure 4 — Effects of Hoagland nutrient solution with different H2O2 concentration on peanut after P. liquidambaris colonization under Fe-deficiency. (A): Effects of Hoagland nutrient with different H2O2 concentration on root fresh weight. (B): Effects of Hoagland nutrient with different H2O2 concentration on shoot fresh weight. (C): Effects of Hoagland nutrient with different H2O2 concentration on total fresh weight. Data and errors are mean ± SD, n = 6, and different letters indicate significant differences among treatments. p < 0.05. E = P. liquidambaris inoculation. -Fe = 2 μmol FeEDTA in Hogland nutrition. [file Image_4.JPEG]

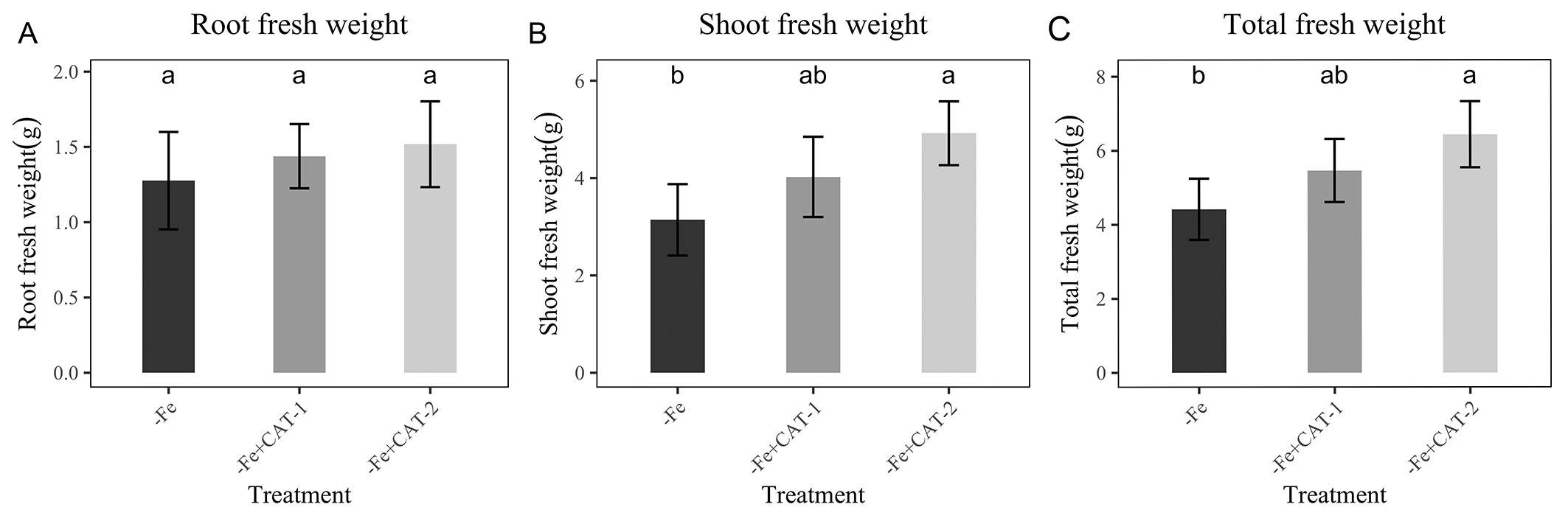

Supplement: Supplementary Figure 5 — Effects of Hoagland nutrient solution with different CAT on peanut under Fe-deficiency. (A): Effects of Hoagland nutrient with different H2O2 concentrations on root fresh weight. (B): Effects of Hoagland nutrient with different H2O2 concentrations on shoot fresh weight. (C): Effects of Hoagland nutrient with different H2O2 concentrations on total fresh weight. Data and errors are mean ± SD, n = 6, and different letters indicate significant differences among treatments. p < 0.05. -Fe = 2 μmol FeEDTA in Hogland nutrition. [file Image_5.JPEG]

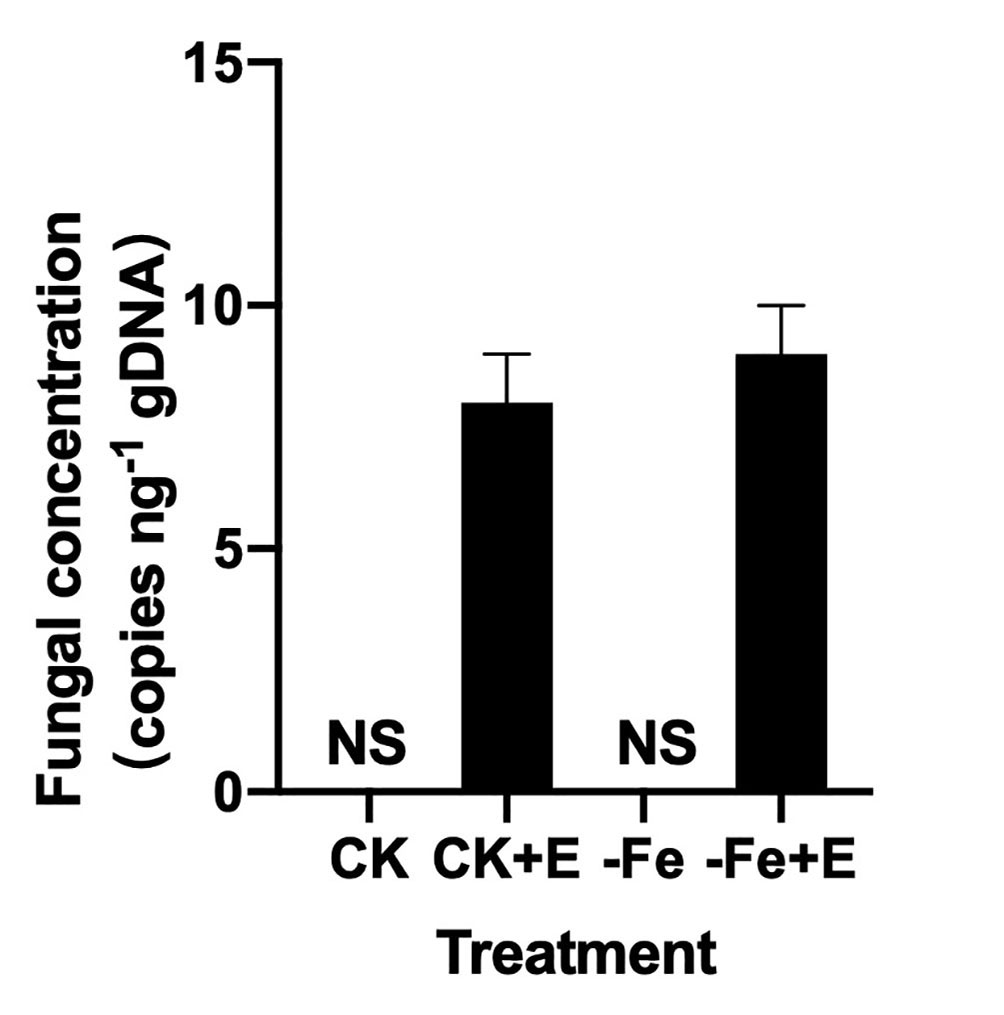

Supplement: Supplementary Figure 6 — Colonization of P. liquidambaris in different treatment groups. The peanuts were transferred to the Hoagland nutrient solution. Seven days later, the colonization was detected by q-PCR. The data shown are the mean ± SD. E =P. liquidambaris inoculation. -Fe = 2 μmol FeEDTA in Hogland nutrition. CK = 100 μmol FeEDTA in Hogland nutrition. [file Image_6.JPEG]

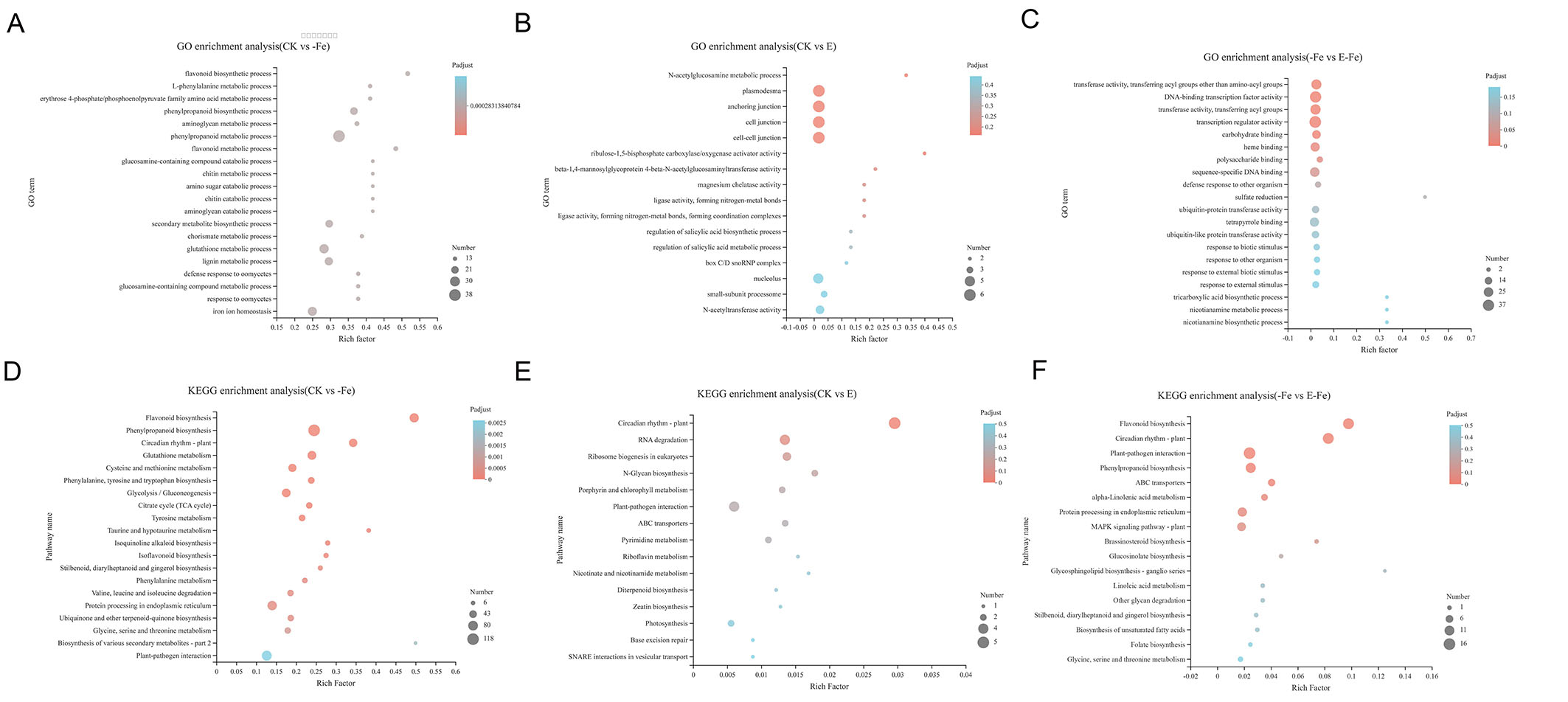

Supplement: Supplementary Figure 7 — GO and KEGG pathway enrichment analysis based on the differentially expressed genes between Fe-sufficient and Fe-deficient peanut roots with or without P. liquidambaris. (A): Go analysis between CK and -Fe. (B): Go analysis between CK and E. (C): Go analysis between -Fe and E-Fe. (D): KEGG analysis between CK and -Fe. (E): KEGG analysis between CK and E. (F): KEGG analysis between -Fe and E-Fe. E = P. liquidambaris inoculation. -Fe = 2 μmol FeEDTA in Hogland nutrition. CK = 100 μmol FeEDTA in Hogland nutrition. [file Image_7.JPEG]

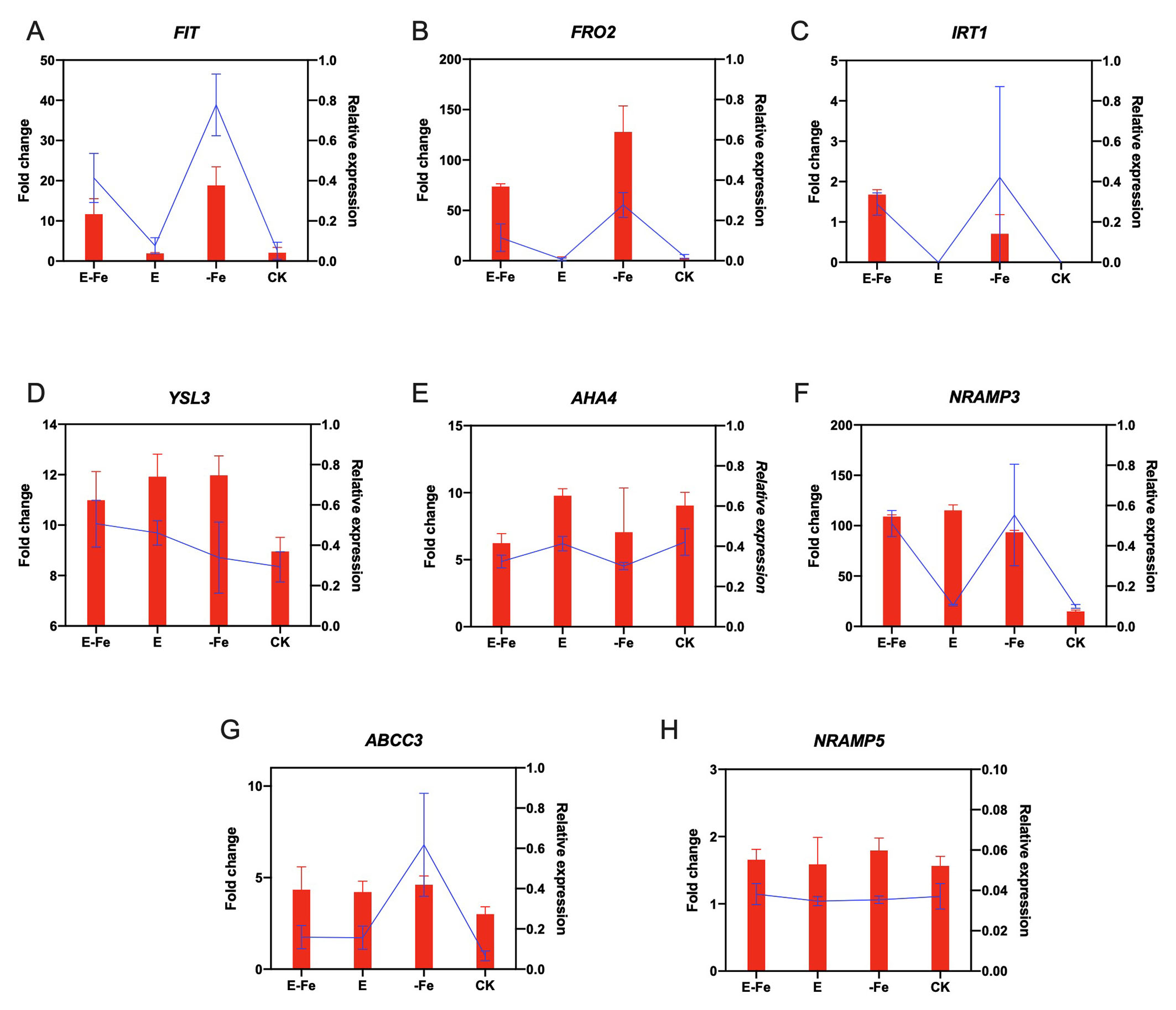

Supplement: Supplementary Figure 8 — The qRT-PCR analysis of genes in the roots under Fe-sufficient or Fe-deficient conditions after P. liquidambaris colonization. The relative expression of each gene was calculated as the 2−ΔΔCT value and normalized to the endogenous reference genes. Data and errors are mean ± SD, n = 6, and different letters indicate significant differences among treatments. p < 0.05. -Fe = 2 μmol FeEDTA in Hogland nutrition. CK = 100 μmol FeEDTA in Hogland nutrition. [file Image_8.JPEG]
