# Supplementary material for: Endophytic Fungus Phomopsis liquidambaris Enhances Fe Absorption in Peanuts by Reducing Hydrogen Peroxide
Source: Front Plant Sci. 2022 Apr 29;13:872242. doi: 10.3389/fpls.2022.872242 (PMC9100952; doi:10.3389/fpls.2022.872242)
Supplement: Supplementary Table 1 — Primers used for RT-qPCR analysis. [file Table_1.DOCX]

**Table S1.** Primers used for RT-qPCR analysis.

| Primer names | Primer sequences(5’-3’) |
| --- | --- |
| *AhFIT-F* | CTT AATTACAATG GATGCTCAC |
| *AhFIT-R* | GATGAATCTGATCATATGGG |
| *AhActin-F* | CTGAAAGATTCCGATGCCCTGA |
| *AhActin-R* | AACCACCACTCAAGACAATGTTACCA |
| *AhIRT1-F* | GTTCTCTGCCTTATTCACGCTCAT |
| *AhIRT1-R* | GCCAACACTAACAACAACACCCAT |
| *AhFRO2-F* | ACTAATATTGGGTTTGGTTGGTAACATA |
| *AhFRO2-R*  *AhAHA4-F*  *AhAHA4-R*  *AhNRAMP1-F*  *AhNRAMP1-R*  *AhFRO1-F*  *AhFRO1-R*  *AhNRAMP3-F*  *AhNRAMP3-R*  *AhNRAMP5-F*  *AhNRAMP5-R*  *AhYSL3-F*  *AhYSL3-R*  *AhABCC3-F*  *AhABCC3-R* | GGTGAGGCCAAGAAGTGGAA  GGGTGAGATAGAAGCAGTTGTGATT  TGGAAGTGTCCAACTTGGTTAGTG  CCTCATCACTGCCTTCGT  ATTGCTGTGTTATCCTTGGTC  GAAACTGGAGGACGCAGGACTAA  ATGGGCAGTGAAGAAAGTGAGAA  AGGTTGAAAAAATGGATGAGAGC  TCAGAACATCTAACGATTGCTCAG  TTACTCCCAAACTCAGTGGTCAAG  GTGGAGGAAGAGGTTGTGCG  TATTTGGAGAACCAGAGGCAGC  CGCCAACGATACTGGAATGC  TTTCTTCGTTCCTTCGTCTCG  TCCCACAAACAGCAACCCTC |
